# Hydrogen-bonded macrocycle-mediated dimerization for orthogonal supramolecular polymerization

**DOI:** 10.3762/bjoc.21.10

**Published:** 2025-01-17

**Authors:** Wentao Yu, Zhiyao Yang, Chengkan Yu, Xiaowei Li, Lihua Yuan

**Affiliations:** 1 College of Chemistry, Sichuan University, Chengdu 610064, China

**Keywords:** hydrogen-bonded macrocycle, orthogonal self-assembly, shape-persistent, supramolecular polymer

## Abstract

Orthogonal self-assembly represents a useful methodology to construct supramolecular polymers with AA- and AB-type monomers, as commonly used for covalently linked polymers. So far, the design of such monomers has relied heavily on three-dimensional macrocycles, and the use of two-dimensional shape-persistent macrocycles for this purpose remains rather rare. Here, we demonstrate a dimerization motif based on a hydrogen-bonded macrocycle that can be effectively applied to form orthogonal supramolecular polymers. The macrocycle-mediated connectivity was confirmed by single-crystal X-ray diﬀraction, which revealed a unique 2:2 binding motif between host and guest, bridged by two cationic pyridinium end groups through π-stacking interactions and other cooperative intermolecular forces. Zinc ion-induced coordination with the macrocycle and a terpyridinium derivative enabled orthogonal polymerization, as revealed by ^1^H NMR, DLS, and TEM techniques. In addition, viscosity measurements showed a transition from oligomers to polymers at the critical polymerization concentration of 17 μM. These polymers were highly concentration-dependent. Establishing this new dimerization motif with shape-persistent H-bonded macrocycles widens the scope of noncovalent building blocks for supramolecular polymers and augurs well for the future development of functional materials.

## Introduction

Host–guest interactions, particularly those involving macrocycles as hosts [1], have found a myriad of applications in supramolecular chemistry [2–4] owing to their ability to create noncovalent, dynamic, yet in some cases strong forces between molecules. Adding an additional element of interactions to supramolecular systems endows them with a feature of “orthogonal self-assembly”, a process in which molecular species are assembled into aggregates by two or more types of noncovalent interactions that work independently from each other [5–8]. Generating such motifs with orthogonal propensity is appealing not only for the construction of supramolecular polymers with the ability to modulate their structure and properties in multiple ways through adjustment of noncovalent bonding interactions. It also confers on supramolecular assemblies with higher complexity and multilevel ordering [9–11], leading to a vast number of applications, for example, for use in detection and separation [12], sensing [13], photocatalysis [14], release [15], and as thermochromic and photoluminescent materials [16]. In this regard, macrocycles emerged a decade ago as a “sticking” end for homo- and heterodifunctional monomers, enabling supramolecular polymerization [17]. So far, macrocycles that are applied to orthogonal self-assembly have been limited to three-dimensional rings, such as cucurbituril [18], cyclodextrin [19], and calix[4]pyrrole [20], as well as flexible crown ethers [10,21]. Few two-dimensional (2D) shape-persistent macrocyclic compounds are used for this purpose. One difficulty in realizing 2D macrocycle-based orthogonal assembly is that the construction motif must be capable of dimerization by binding to a macrocycle in a 2:2 stoichiometry [22–23]. Such binding motifs are intriguing for macrocycle-mediated supramolecular dimerization since they may enable multiple modes of noncovalent connectivity through combination with other noninterfering interactions (e.g., metal coordination interactions and hydrogen bonding), providing access to various multiresponsive orthogonal self-assemblies or smart supramolecular polymers [24–25]. For example, the discovery of cucurbit[8]uril complexation in a 1:2 and 2:2 host–guest stoichiometry leads to a wide spectrum of applications, which includes catalysis [26], gelation [27], sensing [28], color tuning, etc. [29]. However, only several kinds of macrocycles are capable of supramolecular dimerization through host–guest interactions [30].

**Scheme 1:**
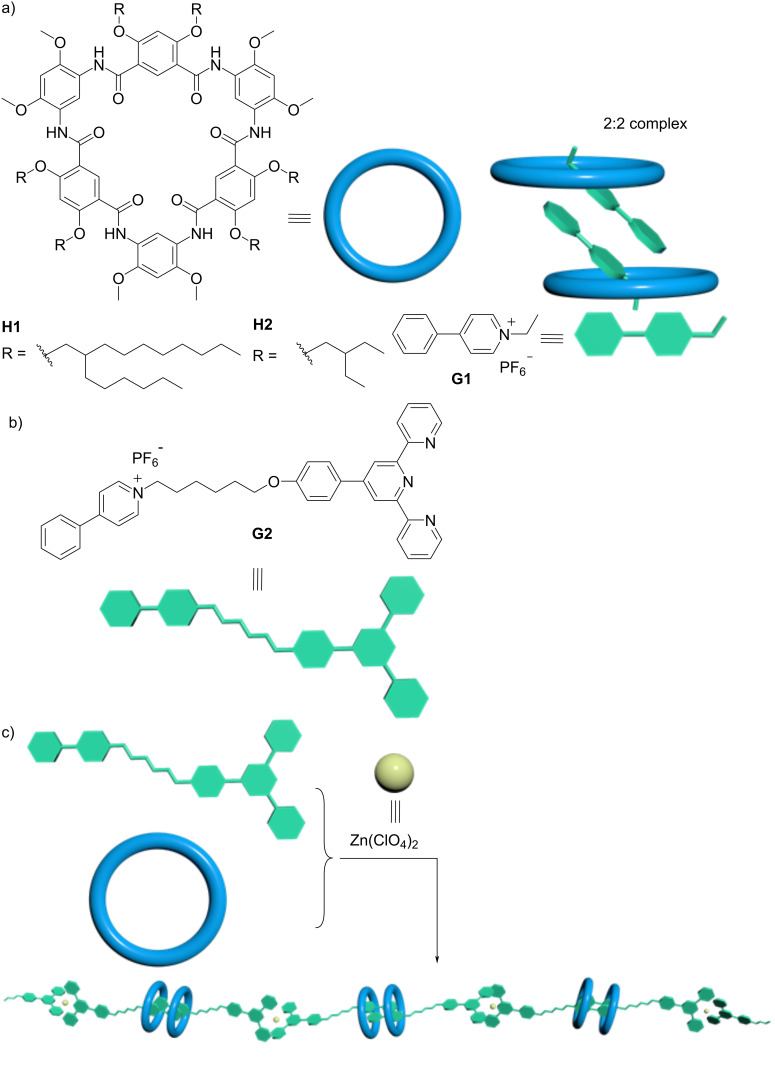
a) Chemical structures of H-bonded macrocycles **H1**, **H2**, and guest **G1**, and schematic representation of the formation of a 2:2 host–guest complex. b) Chemical structure of guest **G2**. c) Schematic representation of the formation of a supramolecular polymer through orthogonal self-assembly upon addition of zinc ions.

Shape-persistent macrocycles have captured the interest of chemists for decades [31–32]. This is not only due to their rich host–guest chemistry but also due to π-surface-enabled self-assembly that enables the creation of various supramolecular structures, such as discotic liquid crystals [33], nanodimers [34], and organic frameworks [35]. Among them, shape-persistent hydrogen-bonded aromatic amide macrocycles [36–42], a class of cyclic compounds comprising a number of aromatic residues with consecutive intramolecular hydrogen bonds and amide linkages, stand out as versatile host molecules as their cavity size, peripheral side chains, and recognition sites are tunable to suit desired functions. These macrocycles have found widespread applications owing to their unique host–guest behaviors in the fields of recognition [43], ion channels [44], catalysis [45], rotaxanes [46], as well as molecular machines [47]. We envisioned that the use of a H-bonded aromatic amide macrocycle with six aromatic residues (hereafter called cyclo[6]aramide for brevity, Scheme 1a) could mediate dimerization as a host. That is because such a 2D macrocycle has six carbonyl oxygen atoms pointing inwards as binding sites, demonstrating excellent affinity for cationic organic guests, including pyridinium and its derivatives [40]. More importantly, it favors intermolecular π-stacking interactions with aromatic guests [48]. Herein, we report on a novel supramolecular dimerization motif in 2:2 stoichiometry using H-bonded aramide macrocycles for constructing orthogonal supramolecular polymers (Scheme 1).

The terpyridyl group and the pyridinium cation in the AB-type monomer **G2** each function as a “sticker” to enable supramolecular polymerization in the presence of the macrocyclic component and zinc ions. The driving force for the recognition involves multiple cooperative interactions, particularly π-stacking interactions between the aromatic rings in a parallel fashion from both host and guest, which is demonstrated by the crystal structure of the key element of the recognition motif.

## Results and Discussion

### Backdrop for design considerations

**Figure 1:**
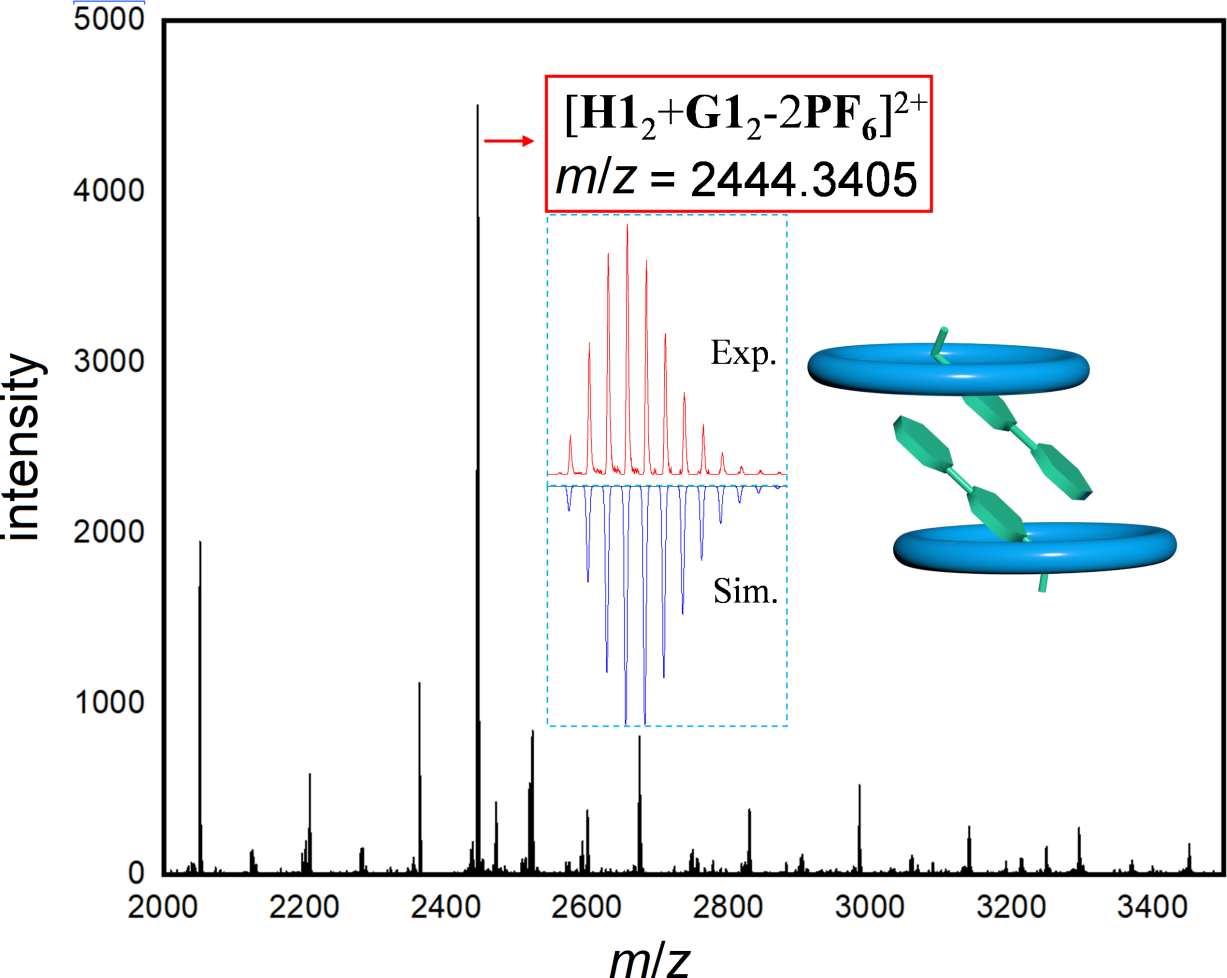
ESIMS spectrum of an equimolar mixture of **G1** and **H1** in CHCl_3_/CH_3_CN (1:1, v/v), including calculated (blue) and experimental (red) isotopic distribution for [**H1**_2_ + **G1**_2_ − 2PF_6_^−^]^2+^. Calculated and found *m*/*z* = 2444.3373 and 2444.3405, respectively.

**Figure 2:**
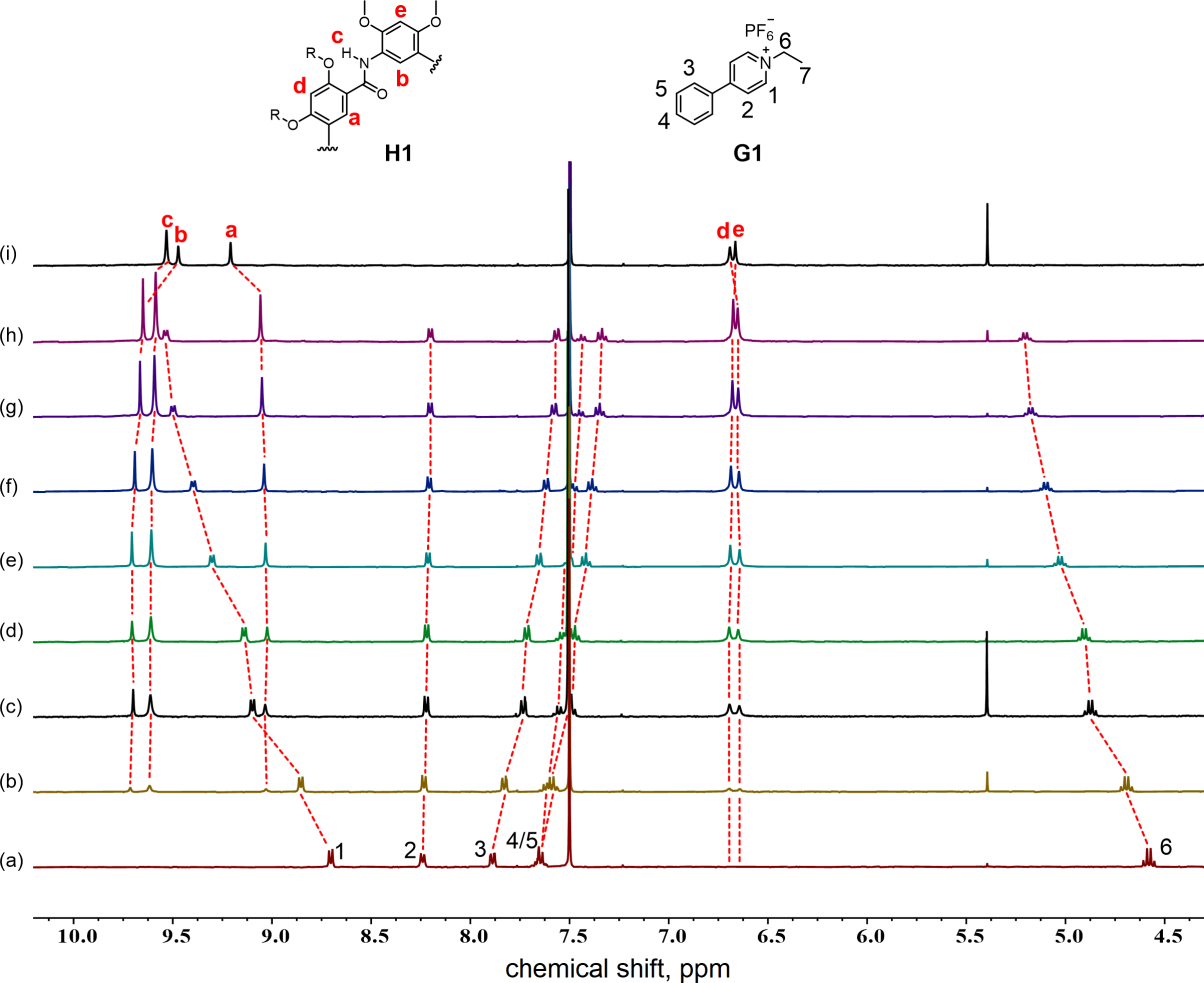
Stacked ^1^H NMR spectra (CDCl_3_/CD_3_CN 1:1, v/v, 400 MHz, 298 K) of **G1** upon addition of different equiv of **H1** ([**G1**] = 1.0⋅10^−3^ M). (a) 0.0 equiv, (b) 0.2 equiv, (c) 0.4 equiv, (d) 0.6 equiv, (e) 0.8 equiv, (f) 1.0 equiv, (g) 1.2 equiv, (h) 1.4 equiv, and (i) only **H1**.

Our interest in macrocycle-mediated supramolecular dimerization was triggered by observations in mass spectrometry experiments when exploring host–guest interactions. Positive-ion electrospray ionization mass spectrometry (ESI(+)MS) of a sample solution of cyclo[6]aramide **H1** and a pyridinium derivative **G1** in CHCl_3_/CH_3_CN (1:1, v/v) showed a dominant peak at *m*/*z* = 2444.3405, corresponding to the cation [**H1**_2_ + **G1**_2_ − 2PF_6_^−^]^2+^, indicating the presence of a host–guest complex in a 2:2 stoichiometry in the gas state (Figure 1). Job plot experiments provided a 1:1 stoichiometry (Figure S9, Supporting Information File 1), showing consistency with the molar ratio observed in ESI experiments. These inspiring results prompted us to further examine the interaction between host **H1** and **G1** by ^1^H NMR spectroscopy. Our prior experience with cyclo[6]aramide has confirmed binding to the cationic guest [49]. Indeed, addition of compound **H1** to the guest solution resulted in a pronounced downfield shift (Δδ = +0.694 ppm) of protons H^1^ relative to the proton resonance of the free guest **G1** in CDCl_3_/CD_3_CN (1:1, v/v, Figure 2). Proton H^6^ also experienced a larger downfield shift (Δδ = +0.519 ppm). The fact that all other resonances for protons H^2^–H^5^ only showed relatively small lower frequency shifts (Δδ = −0.033–0.270 ppm) with respect to protons H^1^ and H^6^ suggested that the macrocycle tends to reside around the cationic pyridinium site in the binding process. On the other hand, the change in the chemical shift of the protons of **G1** was accompanied by a change in the resonance of the internal aromatic protons H^a^ and H^b^ in the host **H** (Δδ= −0.170 and +0.218 ppm, respectively), which all pointed toward the existence of strong host–guest interactions in solution.

**Figure 3:**
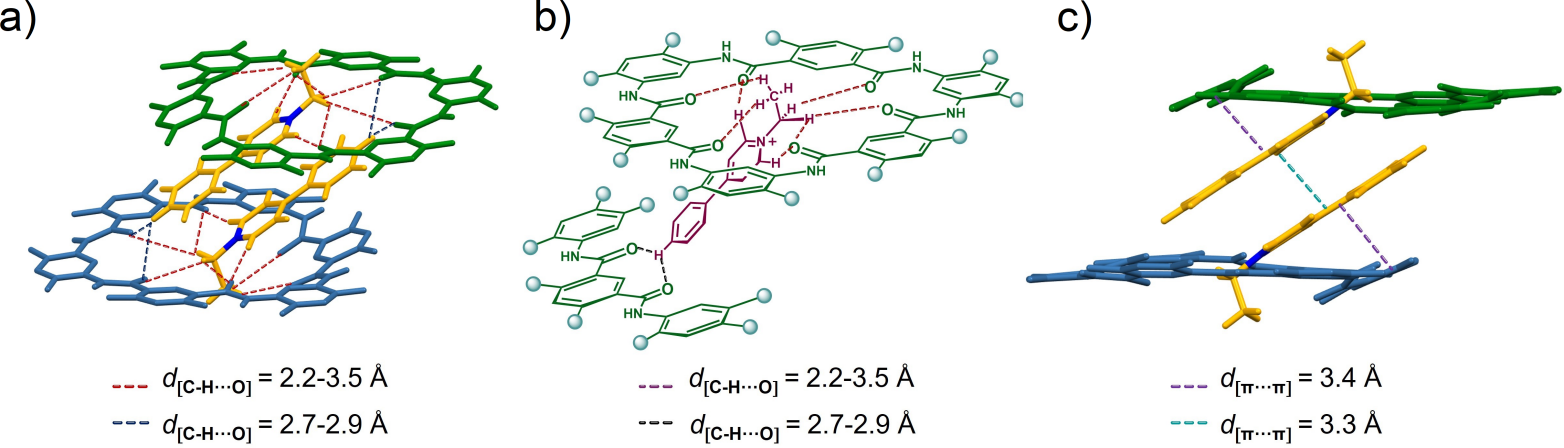
Single-crystal X-ray structure of the complex **H2** ⊃ **G1**. a) Dimeric structure formed by cyclo[6]aramide **H2** and cationic guest **G1**, with each guest molecule threading one molecule of **H2** at its end. b) A portion of the dimeric structure showing an array of hydrogen bonding interactions between the amide oxygen atoms of **H2** and **G1**. The brown dotted lines show the hydrogen bond distance between **H2** and the positively charged region of **G1** (*d*_[C–H···O]_ = 2.2–3.5 Å). The red dotted lines show the hydrogen bond distance between **H2** and the proton of the phenyl group of **G1** (*d*_[C–H···O] _*=* 2.7–2.9 Å). c) Dimeric structure showing the π–π-stacking distances (*d*_[π···π] _*=* 3.3–3.4 Å) between **H2** and **G1**. The side chains of **H2** were replaced by a methyl group, all hydrogen atoms except amide groups, solvent molecules, and counterions were omitted for clarity.

Then we attempted to grow single crystals of the complex out of curiosity, wondering whether the 2:2 structure would also be found in the solid state. Fortunately, single crystals of the complex **H**1 ⊃ **G1** were obtained by slow evaporation of chloroform/acetone solvent (1:1, v/v) into a small amount of methanol over the course of two weeks. Indeed, analysis of the crystal structure of the complex revealed a strict 2:2 molar ratio (Figure 3), providing convincing evidence for the dimerization process. The complex crystallized in the monoclinic *P*2(1)/*c* space group with lattice constants *a* = 24.434(4) Å, *b* = 20.026(3) Å, and *c* = 23.779(4) Å. The dimeric superstructure was stabilized by multiple cooperative noncovalent interactions, particularly intermolecular π–π-stacking and C–H···O interactions. Specifically, π–π-stacking interactions were found between two guest molecules with aromatic rings arrayed in an offset fashion with a distance of 3.3 Å (Figure 3a). Interestingly, these interactions also existed between one guest and one phenyl ring of the host with a distance of 3.4 Å. The observation of these short π-distances suggested the crucial role of π-stacking interactions in sustaining the stability of the 2:2 complex of host **H2** and guest **G1**. It is worth mentioning that each of the two macrocycles adopted a nonplanar conformation, with one aromatic residue protruding out of the macrocyclic skeleton plane. Adopting such a conformation rendered it possible for the π-electron-rich phenyl ring to interact strongly with π-electron-deficient guest molecules **G1** by means of charge–transfer interactions and/or π-stacking interactions, conferring the characteristic 2:2 constitutional stoichiometry onto this host–guest complex. In addition, there were eight C–H···O interactions between the hydrogen atoms of **G1** and the nearby pyridinium group of **H2**, with distances of 2.2–3.5 Å, and two C–H···O interactions between hydrogen atoms of **G1** and the nearby phenyl groups of **H2**, with a distance of 2.7–2.9 Å (Figure 3a and Figure 3b). These results provided conclusive evidence for the 2:2 recognition mode through interaction of two guests with two macrocycles.

With the 2:2 host–guest complexation pattern in mind, a guest molecule **G2** consisting of phenylpyridinium and a terpyridyl group was designed (Scheme 1b) to form a host–guest complex with cyclo[6]aramide **H1**. Compound **G2** was synthesized by refluxing **S2** and 4-phenylpyridine in acetonitrile for 24 h (Scheme S2, Supporting Information File 1). It was expected that by applying the design rules commonly known for metallosupramolecular polymers and macrocycle-mediated dimerization to supramolecular chemistry, we would be able to generate supramolecular polymers with this heterodifunctional monomer, with one end comprising two macrocycles and the other end coordinating metal ions. In such supramolecular polymers, orthogonal host–guest and coordination interactions are responsible for polymerization.

### Host–guest complexation and zinc coordination

When forming supramolecular polymers, an AB-type monomer, i.e., the guest **G2**, is supposed to interact with the macrocycle **H1** and a metal ion via each of two end groups (pyridinium and terpyridyl). As such, host–guest complexation and zinc coordination of the AB-type monomer were investigated.

**Figure 4:**
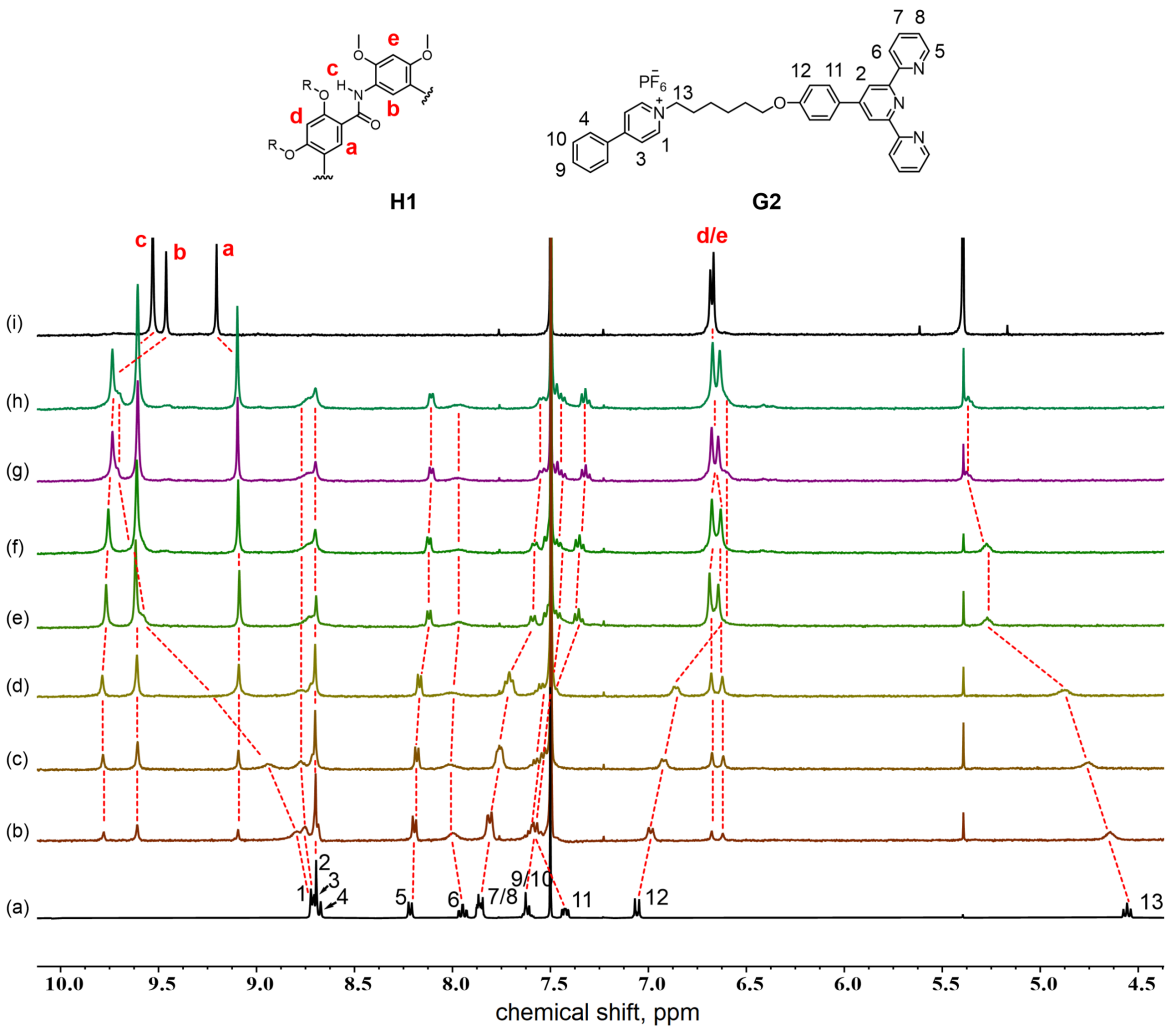
Stacked ^1^H NMR spectra (CDCl_3_/CD_3_CN 1:1, v/v, 400 MHz, 298 K) of **G2** upon addition of different equiv of **H1** ([**G2**] = 1.0⋅10^−3^ M). (a) 0.0 equiv, (b) 0.2 equiv, (c) 0.4 equiv, (d) 0.6 equiv, (e) 0.8 equiv, (f) 1.0 equiv, (g) 1.2 equiv, (h) 1.4 equiv, and (i) only **H1**.

To begin with, the complex formation between host **H1** and **G2** was explored by ^1^H NMR spectroscopic titrations in CDCl_3_/CD_3_CN 1:1, v/v. Titration of compound **H1** to the guest solution resulted in a constant downfield shift of protons H^1^ (Δδ = +0.889 ppm) and H^13^ (Δδ = +0.716 ppm) of the pyridinium moiety, signifying the complexation of the macrocycle at the cationic recognition site. The change in the chemical shift of H^12^ (Δδ = −0.416 ppm) could be explained by ring translocation along the guest axle due to dynamics in the binding process, which is often observed in pseudorotaxanes [50]. When the **H1**/**G2** molar ratio reached 1:1, the original proton signals of the macrocycle disappeared completely (Figure 4). Further addition of the macrocycle marginally influenced the new set of signals, suggesting formation of an *n*:*n* complex of **H1** and **G2**. Job plot experiments afforded a stoichiometry of *n*:*n* (Figure S10, Supporting Information File 1), purporting the probability of 2:2 complexation in solution. A strong indication of the formation of 2:2 complex came from ESIMS experiments. The mass spectra recorded for a 1:1 mixture of **H1** and **G2** showed a multicharged pseudomolecular ion peak corresponding to [**H1**_2_ + **G2**_2_ + H^+^ − 2PF_6_^−^]^3+^, and the isotope patterns were in good agreement with theoretical simulations (Figure S12, Supporting Information File 1). Therefore, taken together, these experimental results indicated that the macrocycle **H1** had a strong propensity for forming a 2:2 stoichiometric complex with guest **G2**.

The ability to coordinate with zinc ions constitutes another important aspect of orthogonal self-assembly. To this end, a mixture of **G2** and zinc salt in CHCl_3_/CH_3_CN (1:1, v/v) was subjected to ^1^H NMR spectroscopy. Protons H^2^–H^4^, H^7^, H^8^, H^11^, and H^12^ of **G2**, located around the metal coordination site, were found to experience upfield or downfield shifts to a varying extent (Figure S8, Supporting Information File 1). Particularly noteworthy was the pronounced downfield shift (Δδ = +0.886 ppm) of the signal of proton H^1^. The change in the chemical shift of the protons of the terpyridyl group suggested the coordination of zinc ion with the terpyridyl end. NOESY data of **H1** ⊃ **G2**, acquired in a CDCl_3_/CD_3_CN solvent mixture (1:1, v/v), disclosed numerous spatial NOE correlations between the internal aromatic protons H^a^–H^c^ of **H1** and the protons H^1^, H^2^, H^5^, H^9^, H^10^, and H^13^ of **G2** (Figure S13, Supporting Information File 1).

### Orthogonal self-assembly and supramolecular polymerization

**Figure 5:**
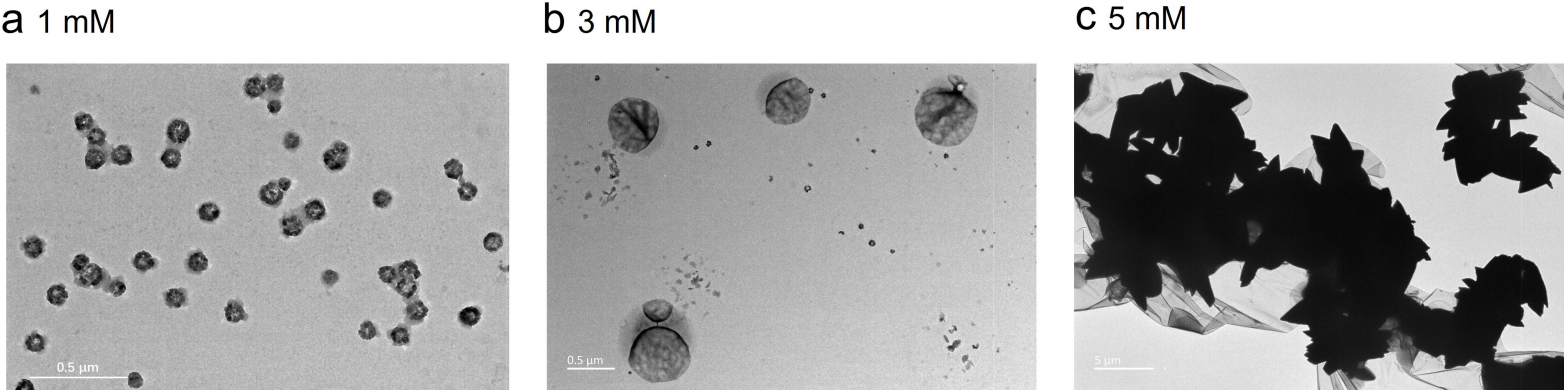
TEM images of a solution of **H1**, **G2**, and Zn(ClO_4_)_2_ at different concentrations (CHCl_3_/CH_3_CN 1:1, v/v, 298 K).

The structure of **G2**, with one end interacting only with macrocycles and the other end coordinating only with metal ions, dictates that self-assembly will occur upon addition of either macrocycle or metal ions. However, supramolecular polymerization is unlikely to be induced unless both are present – a propensity of orthogonal self-assembly. Indeed, when **G2** and zinc salt were mixed together in a 1:1 molar ratio, results from dynamic light scattering experiments showed that the average hydrodynamic diameter (*D*_h_) was 524 nm, and no particles of discernible size were observed for a mixture of the macrocycle **H1** and **G2**, or **H1** and the salt, whereas with a solution containing **G2**, **H1**, and zinc salt, the *D*_h_ of the aggregates abruptly increased to 2580 nm (Figure S15, Supporting Information File 1). These results strongly indicated that polymerization proceeded only when both macrocycles and metal ion were present. TEM images of a solution containing **H1**, **G2**, and Zn^2+^ with a respective concentration of 1 mM and 3 mM revealed a nanoglobular suprastructure, and a larger nanoirregular block suprastructure at higher concentration (5 mM, Figure 5).

**Figure 6:**
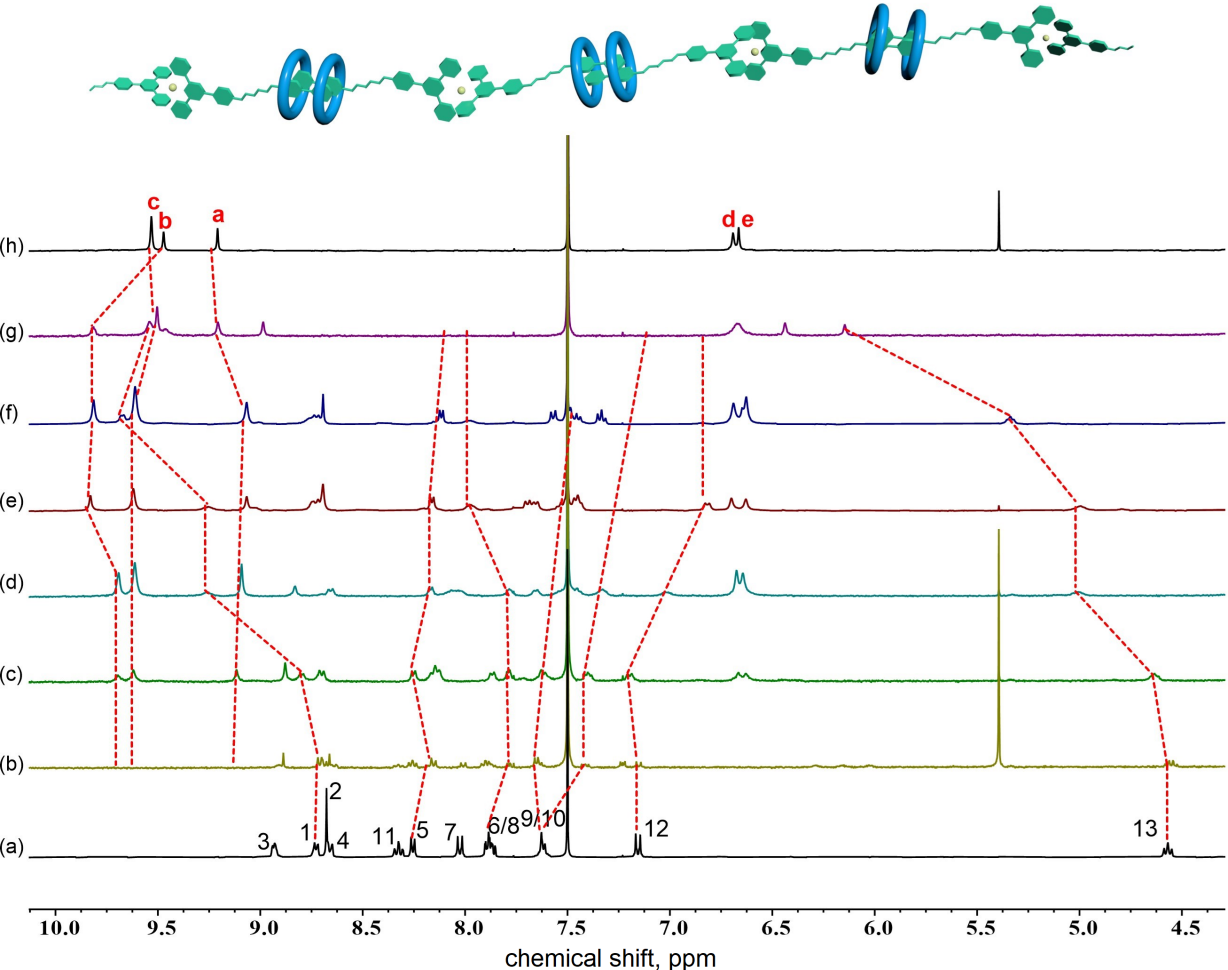
Stacked ^1^H NMR spectra (CDCl_3_/CD_3_CN 1:1, v/v, 400 MHz, 298 K) of **G2** and Zn^2+^ upon addition of different equiv of **H1** ([**G2** + Zn^2+^] = 1.0⋅10^−3^ M). (a) 0.0 equiv, (b) 0.4 equiv, (c) 0.6 equiv, (d) 0.8 equiv, (e) 1.0 equiv, (f) 1.2 equiv, (g) 1.4 equiv, and (h) only **H1**.

To confirm aggregate formation, ^1^H NMR titration experiments were carried out, in which different equiv of the macrocycle **H1** were added to a solution containing a 1:1 mixture of **G2** and zinc salt. Aromatic protons H^a^ (Δδ = −0.142 ppm) and H^b^ (Δδ = +0.357 ppm) were shifted downfield to a great extent, accompanied by the broadening of the signals of **G2** (Figure 6). Constant downfield shifts were observed for protons H^1^ (Δδ = +0.332 ppm) and H^13^ (Δδ = +0.473 ppm) as well as protons H^10^ (Δδ = −0.397 ppm) and H^12^ (Δδ = +0.246 ppm) of the pyridinium moiety, signifying the complexation of the macrocycle at the cationic recognition site. The changes in the chemical shift and the broadening of signals after addition of the macrocycle to a **G2**/Zn^2+^ complex were consistent with the formation of supramolecular polymers.

**Figure 7:**
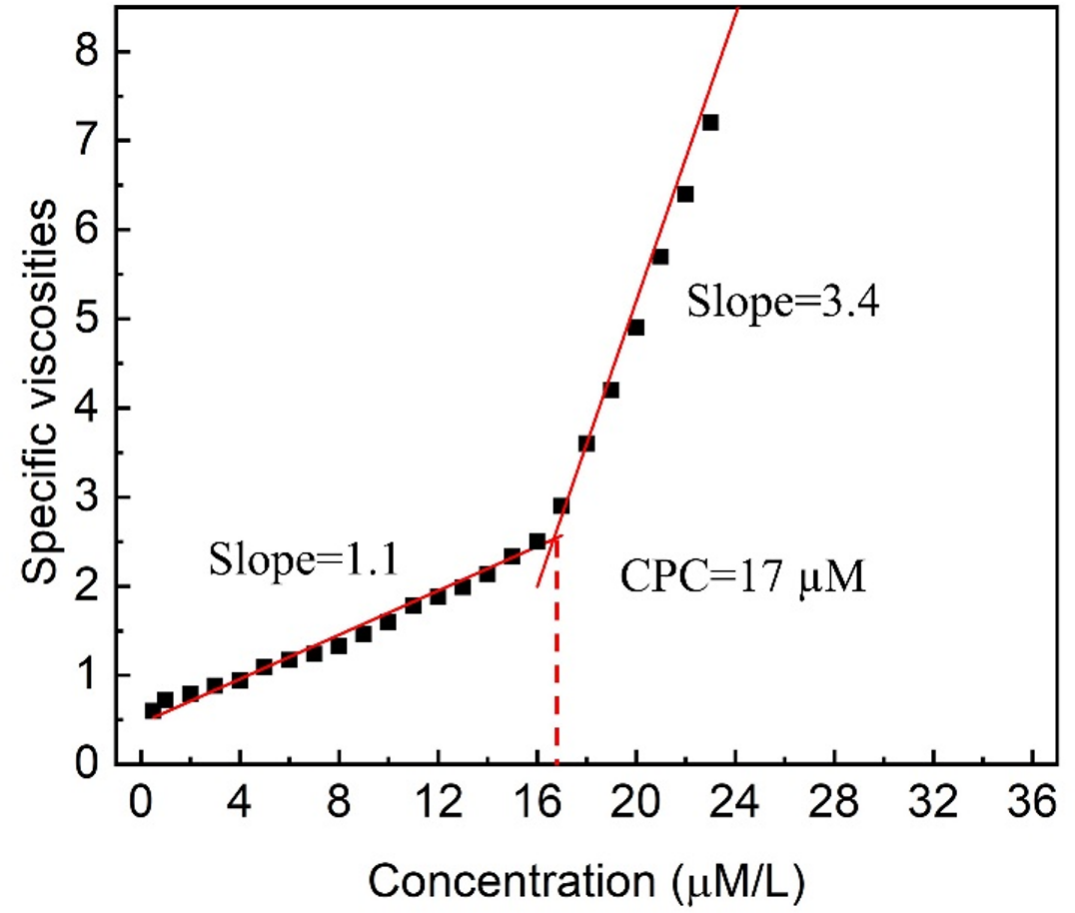
Specific viscosity of the linear supramolecular polymer in CHCl_3_/CH_3_CN (1:1, v/v, 298 K) at variable concentration.

**Figure 8:**
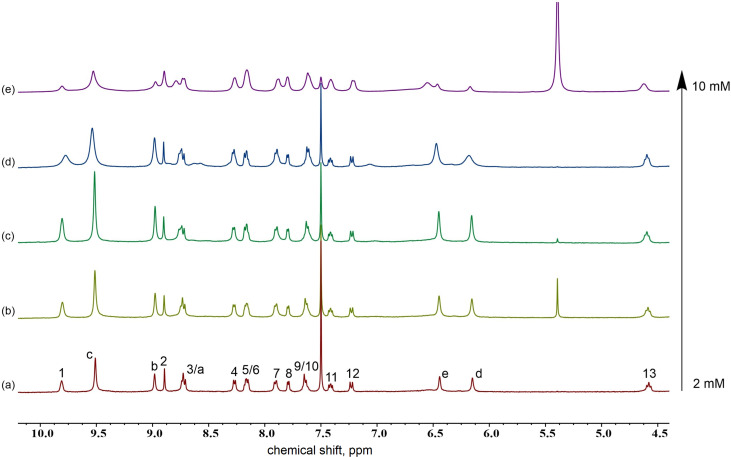
Variable-concentration ^1^H NMR spectra of the supramolecular polymer: (a) 2.0 mM, (b) 4.0 mM, (c) 6.0 mM, (d) 8.0 mM, and (e) 10 mM.

The supramolecular polymers formed were further characterized by viscosity and variable-concentration NMR experiments. One important feature of supramolecular polymers is the dependency of their molecular weight on the solution concentration. Further, a change in solution viscosity would reflect a change in molecular weight during the polymerization process. Thus, the specific viscosity of the linear supramolecular polymer (Scheme 1c) in CHCl_3_/CH_3_CN (1:1, v/v) was determined at 298 K (Figure 7). The specific viscosity was plotted against the concentration, which revealed two stages: prior to the turning point at 17 μM and beyond. The first stage offered a slope of 1.1, corresponding to the formation of oligomeric assemblies in solution. On continuously increasing the concentration beyond 17 μM, a second stage started, with a slope of 3.4, which is usually an indication of the formation of linear supramolecular polymers [51]. The observation of the turning point concentration, or of a critical polymerization concentration (CPC) [52–53], indicated concentration-driven polymerization, which is characteristic for the formation of supramolecular polymers. The concentration-dependency of a mixture of **H1**, **G2**, and zinc salt was examined by variable-concentration NMR spectroscopy. Pronounced broadening of signals was observed (Figure 8), which clearly indicated increasing polymerization upon increasing the concentration of each component.

## Conclusion

We have introduced a new recognition motif based on a hydrogen-bonded aramide macrocycle, which drives the linear polymerization of a heterodifunctional monomer in the presence of zinc ions. In addition to hydrogen bonding interactions, the 2:2 host–guest dimerization is primarily facilitated by enhanced π-stacking interactions between the guests and between the guest and macrocycle. This 2:2 connectivity mode makes shape-persistent H-bonded macrocycles a valuable component for creating orthogonal supramolecular polymers, offering promising potential for designing stimuli-responsive and smart polymers in the future.

## Supporting Information

File 1 CheckCIF report for **H2** ⊃ **G1**.

File 2 Crystallographic data for **H2** ⊃ **G1** analysed with SHELX (CCDC 2384953).

File 3 Experimental data and copies of spectra.

## Data Availability

All data that supports the findings of this study is available in the published article and/or the supporting information of this article.
