# Supplementary material for: Hydrogen-bonded macrocycle-mediated dimerization for orthogonal supramolecular polymerization
Source: Beilstein J Org Chem. 2025 Jan 17;21:179–88. doi: 10.3762/bjoc.21.10 (PMC11744735; doi:10.3762/bjoc.21.10)

BEILSTEIN JOURNAL OF ORGANIC CHEMISTRY

Supporting Information

for

Hydrogen-bonded macrocycle-mediated dimerization for orthogonal supramolecular polymerization

Wentao Yu, Zhiyao Yang, Chengkan Yu, Xiaowei Li and Lihua Yuan

Beilstein J. Org. Chem. **2025,** *21,* 179–188. doi:10.3762/bjoc.21.10

CheckCIF report for H2 ⊃ G1

License and Terms: This is a supporting information file under the terms of the Creative Commons Attribution License (https://creativecommons.org/licenses/by/4.0). Please note that the reuse, redistribution and reproduction in particular requires that the author(s) and source are credited and that individual graphics may be subject to special legal provisions.
The license is subject to the *Beilstein Journal of Organic Chemistry* terms and conditions: (https://www.beilstein-journals.org/bjoc/terms)

checkCIF/PLATON report

Structure factors have been supplied for datablock(s) 4bp

THIS REPORT IS FOR GUIDANCE ONLY. IF USED AS PART OF A REVIEW PROCEDURE FOR PUBLICATION, IT SHOULD NOT REPLACE THE EXPERTISE OF AN EXPERIENCED CRYSTALLOGRAPHIC REFEREE.

No syntax errors found. CIF dictionary Interpreting this report

Datablock: 4bp

```
Bond precision:  C-C = 0.0087 A          Wavelength=0.71073

Cell:            a=24.434(4)     b=20.026(3)         c=23.779(4)
                 alpha=90        beta=118.706(2)     gamma=90
Temperature:     123 K
```

	Calculated	Reported
Volume	10205(3)	10205(3)
Space group	P 21/c	P2(1)/c
Hall group	-P 2ybc	?
Moiety formula	C84 H108 N6 O18, C12 H13 N2, F6 P	?
Sum formula	C96 H121 F6 N8 O18 P	C96 H121 F6 N8 O18 P
Mr	1819.98	1819.98
Dx,g cm-3	1.185	1.185
Z	4	4
Mu (mm-1)	0.103	0.103
F000	3864.0	3864.0
F000’	3866.48	
h,k,lmax	29,23,28	29,23,28
Nref	18170	17763
Tmin,Tmax	0.974,0.982	0.972,0.982
Tmin’	0.972	

```
Correction method= # Reported T Limits: Tmin=0.972 Tmax=0.982
AbsCorr = MULTI-SCAN

Data completeness= 0.978          Theta(max)= 25.100

R(reflections)= 0.1072( 8898)     wR2(reflections)= 0.3153( 17763)

S = 1.173                  Npar= 1263
```

The following ALERTS were generated. Each ALERT has the format
test-name_ALERT_alert-type_alert-level.
Click on the hyperlinks for more details of the test.

Alert level B

```
PLAT220_ALERT_2_B Large Non-Solvent  C     Ueq(max)/Ueq(min) Range        7.1 Ratio
PLAT222_ALERT_3_B Large Non-Solvent  H     Uiso(max)/Uiso(min) ...        8.1 Ratio
PLAT250_ALERT_2_B Large U3/U1 Ratio for Average U(i,j) Tensor ....        4.6 Note
```

Alert level C

```
CRYSC01_ALERT_1_C The word below has not been recognised as a standard
            identifier.
            ?
CRYSC01_ALERT_1_C No recognised colour has been given for crystal colour.
RFACG01_ALERT_3_C  The value of the R factor is > 0.10
            R factor given   0.107
RFACR01_ALERT_3_C  The value of the weighted R factor is > 0.25
            Weighted R factor given   0.315
PLAT029_ALERT_3_C _diffrn_measured_fraction_theta_full Low .......      0.978 Note
PLAT082_ALERT_2_C High R1 Value ..................................       0.11 Report
PLAT084_ALERT_3_C High wR2 Value (i.e. > 0.25) ...................       0.32 Report
PLAT213_ALERT_2_C Atom O1A             has ADP max/min Ratio .....        3.7 prolat
PLAT213_ALERT_2_C Atom C66A            has ADP max/min Ratio .....        3.2 prolat
PLAT220_ALERT_2_C Large Non-Solvent  O     Ueq(max)/Ueq(min) Range        5.4 Ratio
PLAT234_ALERT_4_C Large Hirshfeld Difference O1A    --  C57A    ..       0.17 Ang.
PLAT234_ALERT_4_C Large Hirshfeld Difference C21A   --  C22A    ..       0.18 Ang.
PLAT234_ALERT_4_C Large Hirshfeld Difference C80A   --  C81A    ..       0.18 Ang.
PLAT241_ALERT_2_C High      Ueq as Compared to Neighbors for .....       C25A Check
PLAT241_ALERT_2_C High      Ueq as Compared to Neighbors for .....       C73A Check
PLAT241_ALERT_2_C High      Ueq as Compared to Neighbors for .....         N2 Check
PLAT242_ALERT_2_C Low       Ueq as Compared to Neighbors for .....        O4A Check
PLAT242_ALERT_2_C Low       Ueq as Compared to Neighbors for .....        O5A Check
PLAT242_ALERT_2_C Low       Ueq as Compared to Neighbors for .....        N1A Check
PLAT242_ALERT_2_C Low       Ueq as Compared to Neighbors for .....       C24A Check
PLAT242_ALERT_2_C Low       Ueq as Compared to Neighbors for .....       C57A Check
PLAT242_ALERT_2_C Low       Ueq as Compared to Neighbors for .....       C75A Check
PLAT242_ALERT_2_C Low       Ueq as Compared to Neighbors for .....       C80A Check
PLAT250_ALERT_2_C Large U3/U1 Ratio for Average U(i,j) Tensor ....        3.3 Note
PLAT340_ALERT_3_C Low Bond Precision on  C-C Bonds ..............     0.0087 Ang.
PLAT360_ALERT_2_C Short  C(sp3)-C(sp3) Bond   C81A   -   C82A   ..       1.38 Ang.
PLAT410_ALERT_2_C Short Intra H...H Contact  H5     ..  H9      ..       1.99 Ang.
PLAT412_ALERT_2_C Short Intra XH3 .. XHn     H73A   ..  H78C    ..       1.86 Ang.
PLAT412_ALERT_2_C Short Intra XH3 .. XHn     H75A   ..  H78A    ..       1.88 Ang.
PLAT413_ALERT_2_C Short Inter XH3 .. XHn     H11    ..  H28A    ..       2.01 Ang.
```

Alert level G

```
PLAT002_ALERT_2_G Number of Distance or Angle Restraints on AtSite         22 Note
PLAT003_ALERT_2_G Number of Uiso or Uij Restrained non-H Atoms ...        138 Report
PLAT005_ALERT_5_G No _iucr_refine_instructions_details  in the CIF     Please Do !
PLAT066_ALERT_1_G Predicted and Reported Tmin&Tmax Range Identical          ? Check
PLAT072_ALERT_2_G SHELXL First  Parameter in WGHT Unusually Large.       0.15 Report
PLAT300_ALERT_4_G Atom Site Occupancy of *C77A   is Constrained at      0.500 Check
PLAT300_ALERT_4_G Atom Site Occupancy of *C77B   is Constrained at      0.500 Check
PLAT300_ALERT_4_G Atom Site Occupancy of *C78A   is Constrained at      0.500 Check
PLAT300_ALERT_4_G Atom Site Occupancy of *C78B   is Constrained at      0.500 Check
PLAT300_ALERT_4_G Atom Site Occupancy of *H77A   is Constrained at      0.500 Check
PLAT300_ALERT_4_G Atom Site Occupancy of *H77B   is Constrained at      0.500 Check
PLAT300_ALERT_4_G Atom Site Occupancy of *H77C   is Constrained at      0.500 Check
```

```
PLAT300_ALERT_4_G Atom Site Occupancy of *H77D   is Constrained at      0.500 Check
PLAT300_ALERT_4_G Atom Site Occupancy of *H78A   is Constrained at      0.500 Check
PLAT300_ALERT_4_G Atom Site Occupancy of *H78B   is Constrained at      0.500 Check
PLAT300_ALERT_4_G Atom Site Occupancy of *H78C   is Constrained at      0.500 Check
PLAT300_ALERT_4_G Atom Site Occupancy of *H78D   is Constrained at      0.500 Check
PLAT300_ALERT_4_G Atom Site Occupancy of *H78E   is Constrained at      0.500 Check
PLAT300_ALERT_4_G Atom Site Occupancy of *H78F   is Constrained at      0.500 Check
PLAT300_ALERT_4_G Atom Site Occupancy of *P1     is Constrained at      0.500 Check
PLAT300_ALERT_4_G Atom Site Occupancy of *F1     is Constrained at      0.500 Check
PLAT300_ALERT_4_G Atom Site Occupancy of *F2     is Constrained at      0.500 Check
PLAT300_ALERT_4_G Atom Site Occupancy of *F3     is Constrained at      0.500 Check
PLAT300_ALERT_4_G Atom Site Occupancy of *F4     is Constrained at      0.500 Check
PLAT300_ALERT_4_G Atom Site Occupancy of *F5     is Constrained at      0.500 Check
PLAT300_ALERT_4_G Atom Site Occupancy of *F6     is Constrained at      0.500 Check
PLAT300_ALERT_4_G Atom Site Occupancy of *P1'    is Constrained at      0.500 Check
PLAT300_ALERT_4_G Atom Site Occupancy of *F1'    is Constrained at      0.500 Check
PLAT300_ALERT_4_G Atom Site Occupancy of *F2'    is Constrained at      0.500 Check
PLAT300_ALERT_4_G Atom Site Occupancy of *F3'    is Constrained at      0.500 Check
PLAT300_ALERT_4_G Atom Site Occupancy of *F4'    is Constrained at      0.500 Check
PLAT300_ALERT_4_G Atom Site Occupancy of *F5'    is Constrained at      0.500 Check
PLAT300_ALERT_4_G Atom Site Occupancy of *F6'    is Constrained at      0.500 Check
PLAT301_ALERT_3_G Main Residue  Disorder ........... Percentage =          2 Note
PLAT302_ALERT_4_G Anion/Solvent Disorder ........... Percentage =        100 Note
PLAT304_ALERT_4_G Non-Integer Number of Atoms (   3.50) in Resd. #         3 Check
PLAT304_ALERT_4_G Non-Integer Number of Atoms (   3.50) in Resd. #         4 Check
PLAT432_ALERT_2_G Short Inter X...Y Contact  F1'    ..  C66A    ..       2.68 Ang.
PLAT432_ALERT_2_G Short Inter X...Y Contact  F4'    ..  C25A    ..       2.92 Ang.
PLAT432_ALERT_2_G Short Inter X...Y Contact  O10A   ..  C4      ..       2.98 Ang.
PLAT432_ALERT_2_G Short Inter X...Y Contact  O13A   ..  C10A    ..       3.01 Ang.
PLAT606_ALERT_4_G VERY LARGE Solvent Accessible VOID(S) in Structure        ! Info
PLAT720_ALERT_4_G Number of Unusual/Non-Standard Labels ..........         3 Note
PLAT779_ALERT_4_G Suspect or Irrelevant (Bond) Angle in CIF .... #       356 Check
              C77A -C74A -C77B    1.555   1.555   1.555            12.00 Deg.
PLAT860_ALERT_3_G Number of Least-Squares Restraints .............      2166 Note
PLAT869_ALERT_4_G ALERTS Related to the use of SQUEEZE Suppressed           ! Info
PLAT899_ALERT_4_G SHELXL97   is Deprecated and Succeeded by SHELXL      2014 Note
```

---

0 **ALERT level A** = Most likely a serious problem - resolve or explain
3 **ALERT level B** = A potentially serious problem, consider carefully
30 **ALERT level C** = Check. Ensure it is not caused by an omission or oversight
47 **ALERT level G** = General information/check it is not something unexpected

3 ALERT type 1 CIF construction/syntax error, inconsistent or missing data
29 ALERT type 2 Indicator that the structure model may be wrong or deficient
8 ALERT type 3 Indicator that the structure quality may be low
39 ALERT type 4 Improvement, methodology, query or suggestion
1 ALERT type 5 Informative message, check

It is advisable to attempt to resolve as many as possible of the alerts in all categories. Often the minor alerts point to easily fixed oversights, errors and omissions in your CIF or refinement strategy, so attention to these fine details can be worthwhile. In order to resolve some of the more serious problems it may be necessary to carry out additional measurements or structure refinements. However, the purpose of your study may justify the reported deviations and the more serious of these should normally be commented upon in the discussion or experimental section of a paper or in the "special_details" fields of the CIF. checkCIF was carefully designed to identify outliers and unusual parameters, but every test has its limitations and alerts that are not important in a particular case may appear. Conversely, the absence of alerts does not guarantee there are no aspects of the results needing attention. It is up to the individual to critically assess their own results and, if necessary, seek expert advice.

Publication of your CIF in IUCr journals

A basic structural check has been run on your CIF. These basic checks will be run on all CIFs submitted for publication in IUCr journals (*Acta Crystallographica*, *Journal of Applied Crystallography*, *Journal of Synchrotron Radiation*); however, if you intend to submit to *Acta Crystallographica Section C* or *E*, you should make sure that full publication checks are run on the final version of your CIF prior to submission.

Publication of your CIF in other journals

Please refer to the *Notes for Authors* of the relevant journal for any special instructions relating to CIF submission.

PLATON version of 21/06/2015; check.def file version of 21/06/2015

Datablock 4bp - ellipsoid plot

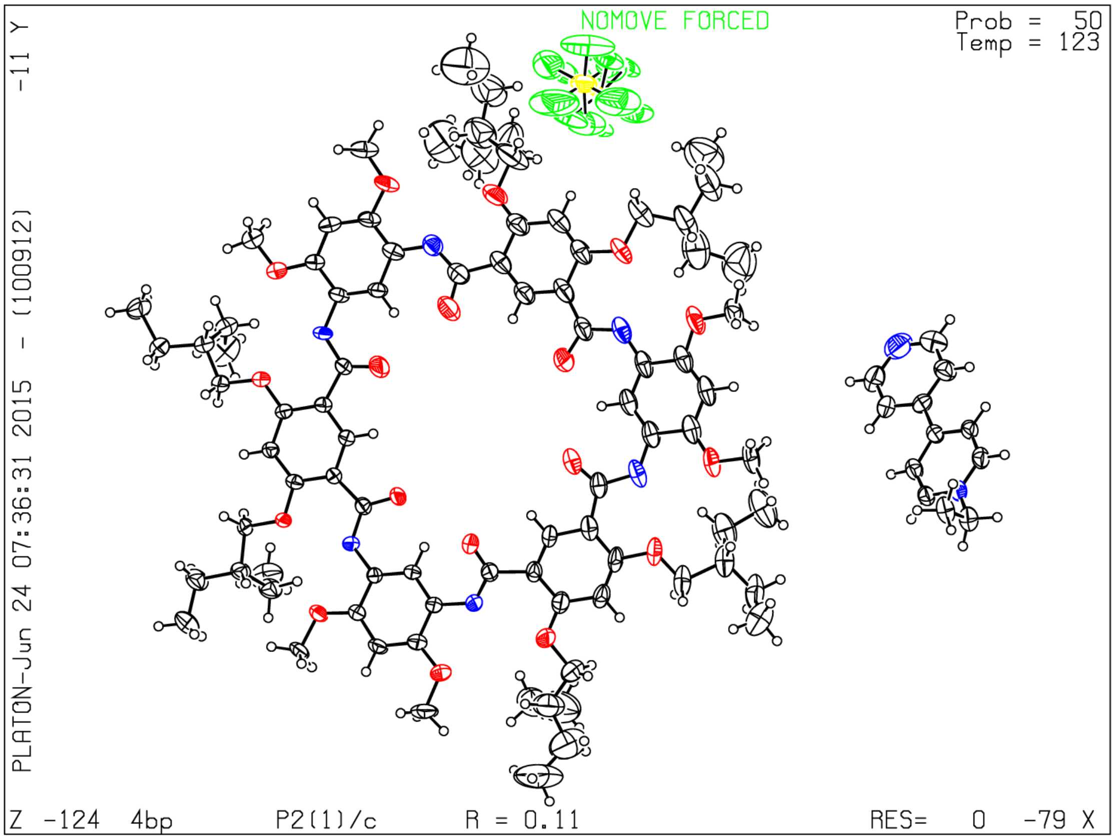

Supplement: File 1 — CheckCIF report for H2 ⊃ G1. [file Beilstein_J_Org_Chem-21-179-s001.pdf]
