# Supplementary material for: Hydrogen-bonded macrocycle-mediated dimerization for orthogonal supramolecular polymerization
Source: Beilstein J Org Chem. 2025 Jan 17;21:179–88. doi: 10.3762/bjoc.21.10 (PMC11744735; doi:10.3762/bjoc.21.10)
Supplement: File 3 — Experimental data and copies of spectra. [file Beilstein_J_Org_Chem-21-179-s003.pdf]

BEILSTEIN JOURNAL OF ORGANIC CHEMISTRY

Supporting Information

for

Hydrogen-bonded macrocycle-mediated dimerization for orthogonal supramolecular polymerization

Wentao Yu, Zhiyao Yang, Chengkan Yu, Xiaowei Li and Lihua Yuan

Beilstein J. Org. Chem. **2025,** *21,* 179–188. doi:10.3762/bjoc.21.10

Experimental data and copies of spectra

License and Terms: This is a supporting information file under the terms of the Creative Commons Attribution License (https://creativecommons.org/licenses/by/4.0). Please note that the reuse, redistribution and reproduction in particular requires that the author(s) and source are credited and that individual graphics may be subject to special legal provisions.
The license is subject to the *Beilstein Journal of Organic Chemistry* terms and conditions: (https://www.beilstein-journals.org/bjoc/terms)

Table of contents

Materials and methods S2

Synthesis and characterization S3

Stacked ^{1}H NMR spectra for **H1** and **G1** interactions.. S7

Stacked ^{1}H NMR spectra for **H1** and **G2** interactions.. S8

^{1}H NMR spectra for **G2** and $Zn(ClO_4)_2$ interactions S9

Job plot for the determination of stoichiometry of host–guest complexes........................... S9

UV–vis titration experiments of macrocycle **H1** and guest **G2**... S10

HRMS spectra of host–guest complexes **H1** + **G2** S11

2D NOESY NMR of the **H1** ⊃ **G2** complex S11

X-ray crystal structure of **G1** ⊂ **H2** S12

DLS data of **G2** + $Zn(ClO_4)_2$ and **H1** + **G2** + $Zn(ClO_4)_2$ S13

TEM of **H1** + **G2** + $Zn(ClO_4)_2$ at variable concentration... S13

Materials and methods

1. Reactions and purifications

All chemicals were obtained from commercial suppliers and used as received unless otherwise noted. All reactions were conducted with oven-dried glassware under ambient atmosphere or nitrogen with stirring. Solvents were dried and distilled according to standard protocols. The reaction progress was monitored using thin-layer chromatography (TLC). Workup and purification procedures were performed with reagent-grade solvents under ambient conditions. Purification via column chromatography was conducted using silica gel (300–400 mesh). Deuterated solvents for NMR experiments were purchased from Energy Chemical.

2. Characterizations

Analytical NMR spectra were recorded on a Bruker AVANCE AV II-400/600 MHz spectrometer at a constant temperature of 298 K. The chemical shift δ is reported in ppm, using tetramethylsilane (TMS) or the residual undeuterated solvent as an internal standard, and coupling constants *J* are denoted in Hz. Multiplicities are indicated as follows: s = singlet, d = doublet, t = triplet, dd = double doublet, m = multiplet, and br = broad. Two-dimensional ROESY NMR spectra were recorded on a Bruker AVANCE AV II-600 MHz spectrometer at 298 K with a 0.4 s mixing time.

UV–vis data were collected on a SHIMADZU UV-2600i spectrometer using HPLC-grade solvents.

ESIMS data were collected on an AB SCIEX X500R spectrometer.

Single-crystal X-ray data were measured on an Xcalibur E diffractometer with graphite monochromated Mo *K*α radiation (λ = 0.71073). Data collection and structure refinement details can be found in the CIF files or obtained free of charge via https://www.ccdc.cam.ac.uk/.

Synthesis and characterization

All of these compounds were prepared following similar procedures reported before. [1-4]

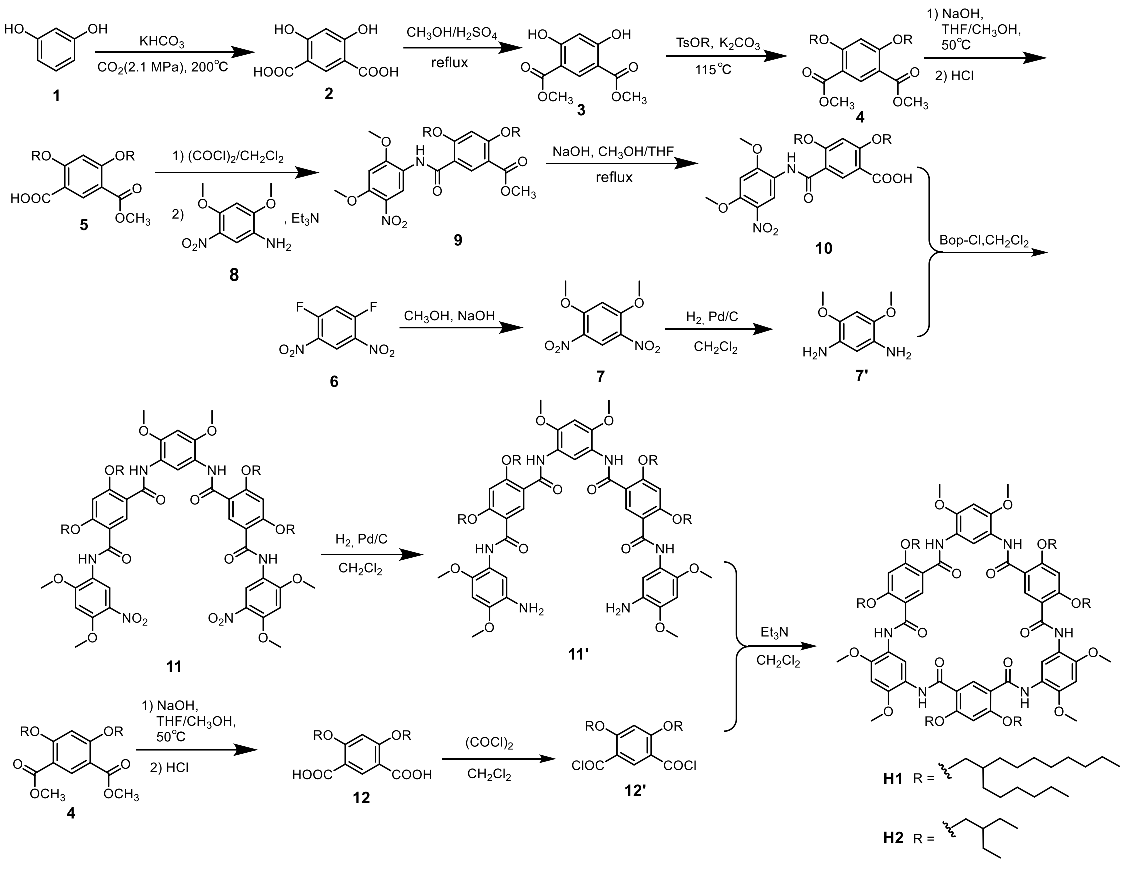

Scheme S1 Synthetic route of **H1** and **H2**.

H1 and **H2** was prepared according to literature procedures. [1]

H1 [1]: white solid powder (yield: 64 %). ^{1}H NMR (400 MHz, $CDCl_3$, 298 K): δ 9.98 (s, 3H), 9.75 (s, 3H), 9.24 (d, *J* = 2.6 Hz, 6H), 7.99 (dd, *J* = 8.7, 2.7 Hz, 3H), 7.09 (d, *J* = 8.8 Hz, 3H), 4.15 (d, *J* = 6.3 Hz, 12H), 3.91 (s, 18H), 2.12 (p, *J* = 6.2 Hz, 6H), 1.63–1.15 (m, 144H), 0.85 (tt, *J* = 6.8, 3.3 Hz, 36H). MADLI-TOF MS m/z calculated for $C_{144}H_{234}N_6O_{18}$ $[M+H]^+$ 2337.7682, found 2337.4220.

H2 [1]: white solid powder (yield: 61 %).^{1}H NMR (400 MHz, $CDCl_3$, 298 K) δ 9.73 – 9.66 (m, 3H), 9.63 – 9.55 (m, 6H), 9.20 (dd, *J* = 5.2, 2.4 Hz, 3H), 6.61 (t, *J* = 3.3 Hz, 6H), 4.20 (d, *J* = 5.0 Hz, 12H), 3.95 (dd, *J* = 3.8, 1.7 Hz, 18H), 2.21 (m, 6H), 1.63 (q, *J* = 6.8 Hz, 24H), 1.03 (m, *J* = 7.9, 2.9, 2.3 Hz, 36H). MADLI-TOF MS m/z calculated for $C_{84}H_{114}N_6O_{18}$ $[M+H]^+$ 1495.826, found 1495.890.

(1) 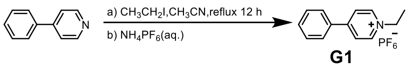

G1

(2) 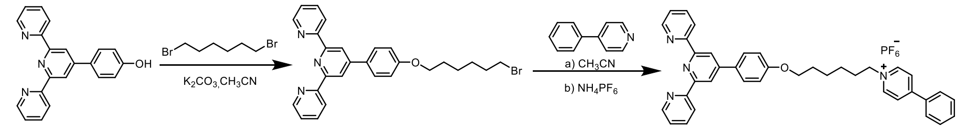

S1 S2 G2

Scheme S2 Synthetic routes of guests **G1** and **G2**.

Guest **G1**[2]: Synthesis of **G1**: A mixture of 4-phenylpyridine (2.00 g, 4.80 mmol), ethyl iodide (2 mL, 6.00 mmol) in 2 mL of acetonitrile was stirred at 90 °C for 24 h. After the reaction solution was cooled down, the solvent was concentrated, and washed with ethyl ether to obtain a white solid powder with a yield of 90%. Ion exchange: dissolve the white solid in 5 mL of methanol, gradually add saturated aqueous solution of NH_4PF_6, precipitate white solid powder, filter and dry to obtain the compound **G1** whose counter ion is hexafluorophosphate. ^{1}H NMR (400 MHz, CD_3OD, 298 K) δ 8.99 (d, J = 7.1 Hz, 2H), 8.41 (d, J = 6.9 Hz, 2H), 8.01 (dd, J = 6.7, 2.9 Hz, 2H), 7.68 – 7.61 (m, 3H), 4.69 (q, J = 7.3 Hz, 2H), 1.69 (t, J = 7.3 Hz, 3H). HRMS (ESI-TOF) m/z: M^+ Calcd for $C_{13}H_{14}PF_6$ 184.1121; Found: 184.1124.

Compound **S2** [3]: Synthesis of **S2**: A mixture of **S1** (500 mg, 1.54 mmol), 1,6-dibromohexane (1.12 g, 4.61 mmol), potassium carbonate (510 mg, 3.69 mmol) in 180 mL of acetonitrile was stirred at 100 °C for 24 h. The reaction solution was cooled down, and the solvent was concentrated. After the reaction solution was cooled, the solvent was concentrated, the precipitate was filtered and washed with acetonitrile for three times (3 × 25 mL), a white solid powder was obtained with 80% yield. ^{1}H NMR (400 MHz, Acetone-d_6, 298 K) δ 8.80 (s, 2H), 8.75 (d, J = 1.5 Hz, 2H), 8.75 – 8.73 (m, 2H), 8.00 (td, J = 7.7, 1.8 Hz, 2H), 7.93 – 7.90 (m, 2H), 7.50 – 7.46 (m, 2H), 7.19 – 7.16 (m, 2H), 4.13 (t, J = 6.4 Hz, 2H), 3.54 (t, J = 6.8 Hz, 2H), 1.94 (d, J = 7.0 Hz, 2H), 1.86 (d, J = 6.7 Hz, 2H), 1.57 (p, J = 3.5 Hz, 4H). HRMS (ESI-TOF) m/z: M^+ Calcd for $C_{27}H_{26}BrN_3O$ 488.1338; Found: 488.1331.

Guest **G2** [4]: Synthesis of **G2**: A mixture of **S2** (300 mg, 614 µmol), 4-phenylpyridine (95.3 mg, 614 µmol) in 10 mL of acetonitrile was stirred at 110 °C for 24 h. A white solid was obtained by filtration under reduced pressure, and the white solid powder was obtained by washing with ether in a yield of 85%.

Ion exchange: The solid was dissolved in CH_3CN/H_2O (1:1, *v/v*) and saturated aqueous NH_4PF_6 was added. The organic solvent was then evaporated under reduced pressure. The precipitate was collected and washed with H_2O, filter and dry to get the compound **G2** with hexafluorophosphate as the counter ion. ^{1}H NMR (400 MHz, $CDCl_3$, 298 K) δ 8.73 (d, J = 4.3

Hz, 2H), 8.69 (s, 3H), 8.65 (d, *J* = 7.0 Hz, 3H), 8.20 (s, 2H), 7.98 (d, *J* = 6.8 Hz, 2H), 7.86 (d, *J* = 7.0 Hz, 4H), 7.62 (d, *J* = 6.9 Hz, 3H), 7.47 – 7.41 (m, 2H), 7.05 (s, 2H), 4.52 (d, *J* = 7.4 Hz, 2H), 4.06 (d, *J* = 6.3 Hz, 2H), 2.06 (d, *J* = 7.6 Hz, 2H), 1.85 (t, *J* = 7.2 Hz, 2H), 1.59 (d, *J* = 7.8 Hz, 2H), 1.48 (d, *J* = 7.5 Hz, 2H). ^{13}C NMR (101 MHz, $CDCl_3$, 298 K) δ 149.12, 137.52, 132.29, 130.10, 129.79, 128.39, 128.03, 125.12, 124.39, 120.58, 118.25, 115.18, 67.31, 48.34, 31.08, 28.44, 25.12, 25.02, 9.24. HRMS (ESI-TOF) m/z: M$^+$ Calcd for $C_{38}H_{35}N_4O^+$ 563.2805; Found: 563.3245.

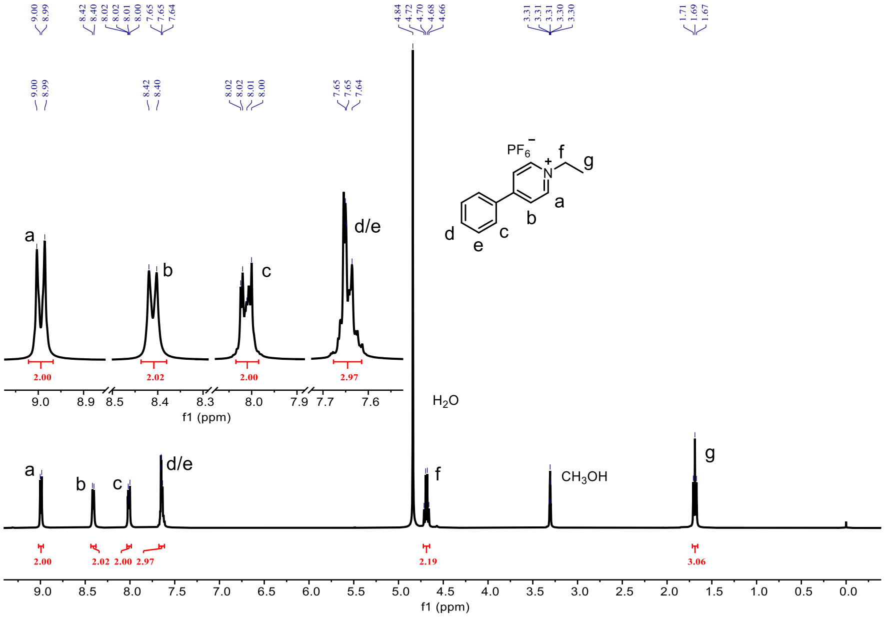

Figure S1 ^{1}H NMR spectrum (400 MHz, CD_3OD, 298 K) of **G1**.

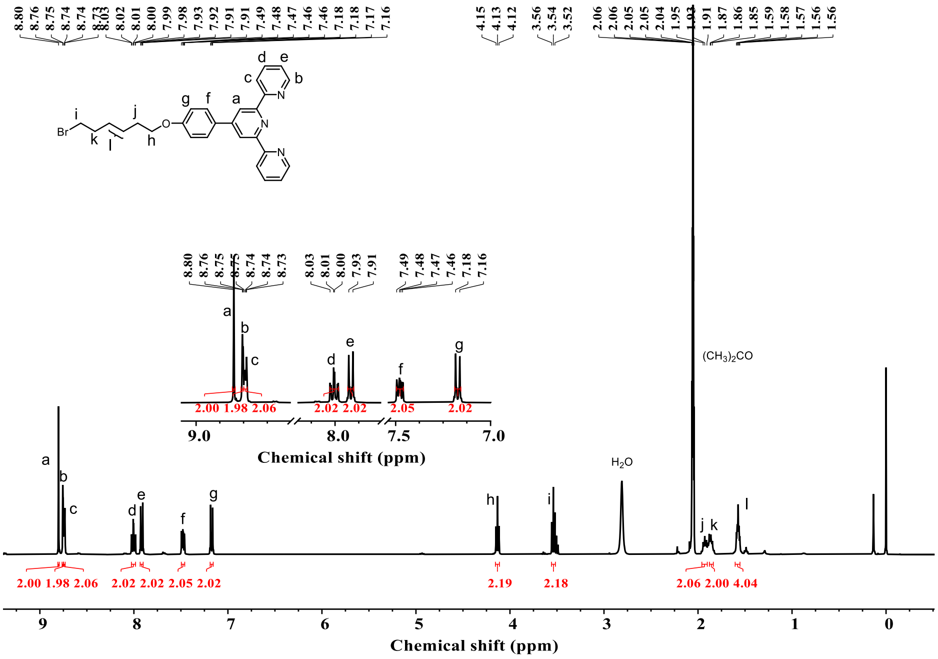

Figure S2 ^{1}H NMR spectrum (400 MHz, Acetone-d_6, 298 K) of **S2**.

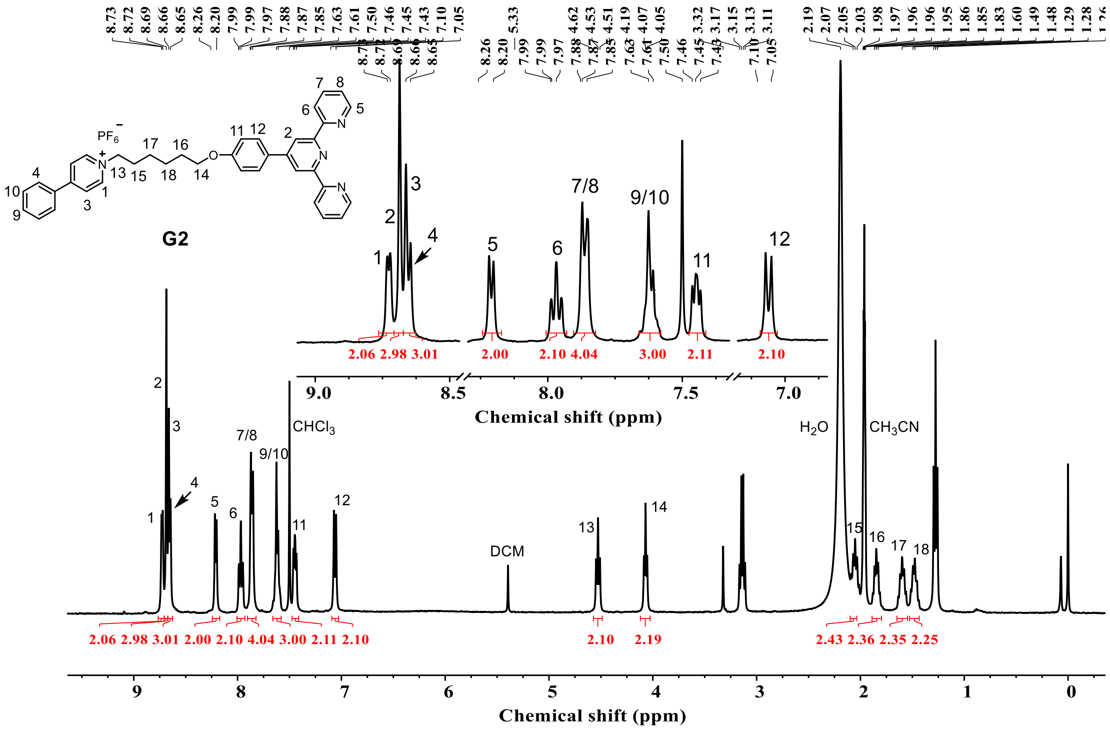

Figure S3 ^{1}H NMR spectrum (400 MHz, $CDCl_3$, 298 K) of **G2**.

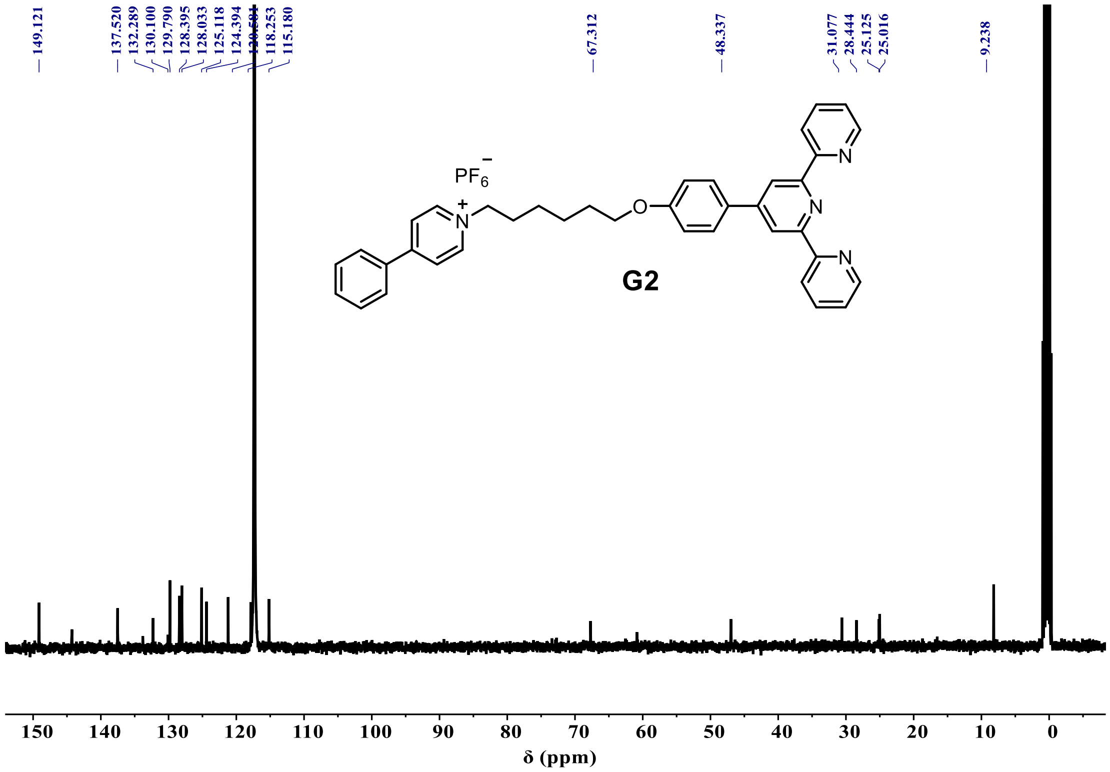

Figure S4 ^{13}C NMR spectrum of **G2** (101 MHz, CD_3CN, 298 K).

Stacked ^{1}H NMR spectra for H1 and G1 interactions

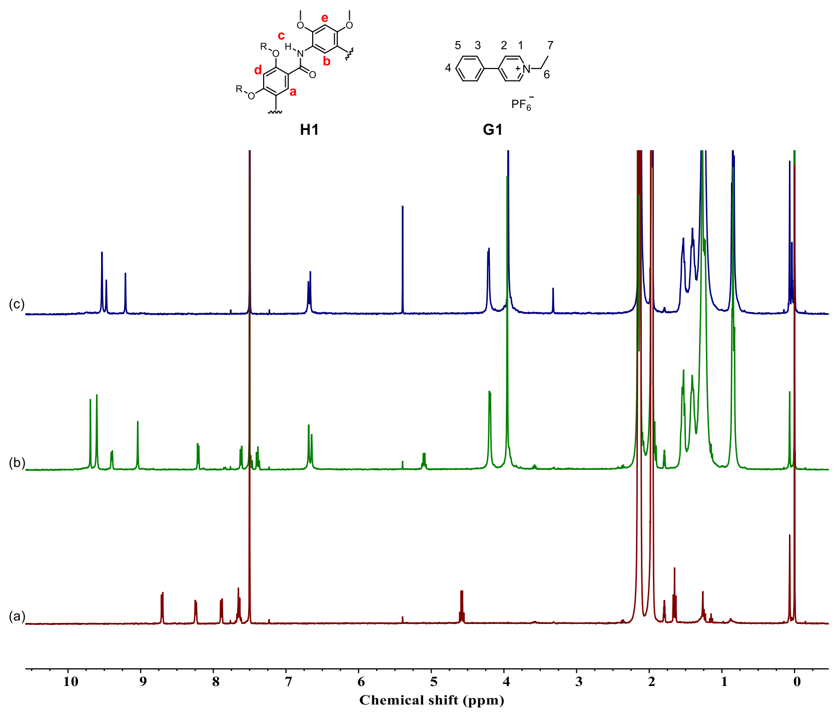

Figure S5 Stacked ^{1}H NMR spectra of (a) **G1**; (b) **H1**: **G1**= 1:1; (c) **H1**. (400 MHz, $CDCl_3/CD_3CN$ = 1:1, v/v, 298 K). [**H1**] = [**G1**] = 1.0 mM.

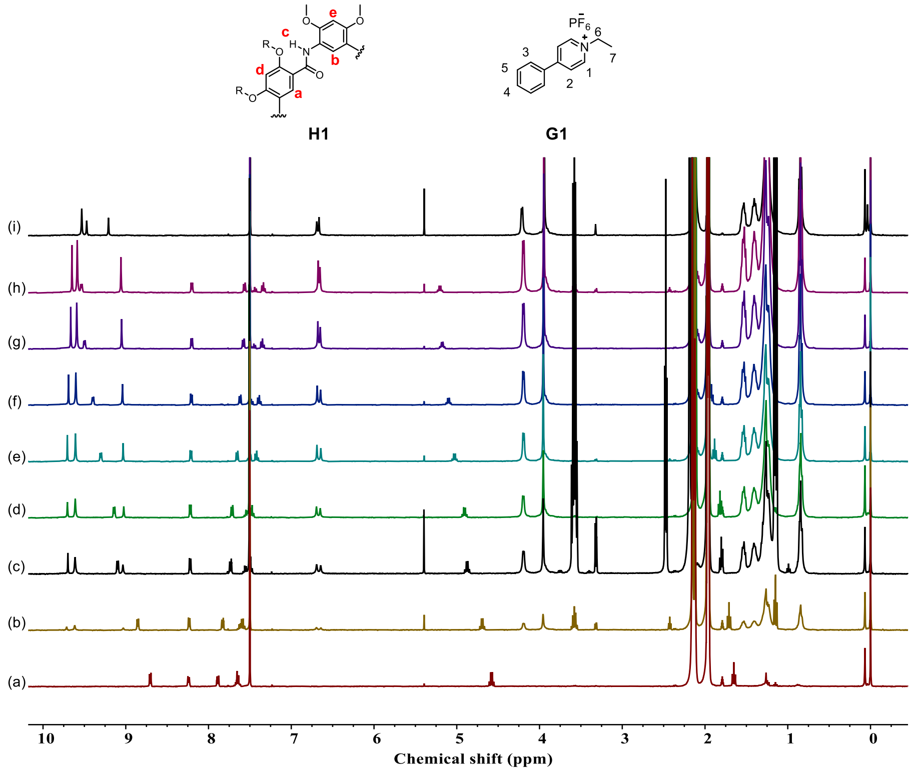

Figure S6 Stacked ^{1}H NMR spectra ($CDCl_3/CD_3CN$= 1:1, v/v, 400 MHz, 298 K) of **G1** upon addition of different equivalents of **H1** ([**G1**] = 1.0 × 10^{-3} M, [**H1**]/[**G1**] = 0–1.4 equiv). (a) 0.0

equiv, (b) 0.2 equiv, (c) 0.4 equiv, (d) 0.6 equiv, (e) 0.8 equiv, (f) 1.0 equiv, (g) 1.2 equiv, (h) 1.4 equiv, and (i) only **H1**.

Stacked ^{1}H NMR spectra for H1 and G2 interactions

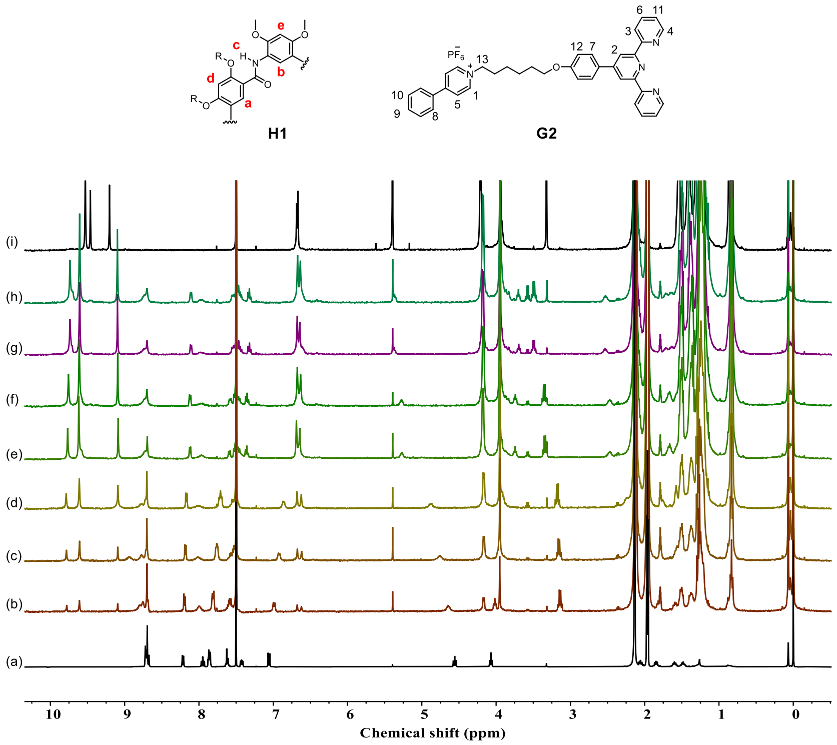

Figure S7 Stacked ^{1}H NMR spectra ($CDCl_3/CD_3CN$= 1:1, v/v, 400 MHz, 298 K) of **G2** upon addition of different equivalent of **H1** ([**G2**] = 1.0 × 10^{-3} M, [**H1**]/[**G2**] = 0–1.4 equiv). (a) 0.0 equiv, (b) 0.2 equiv, (c) 0.4 equiv, (d) 0.6 equiv, (e) 0.8 equiv, (f) 1.0 equiv, (g) 1.2 equiv, (h) 1.4 equiv, and (i) only **H1**.

^{1}H NMR spectra for G2 and $Zn(ClO_4)_2$ interactions

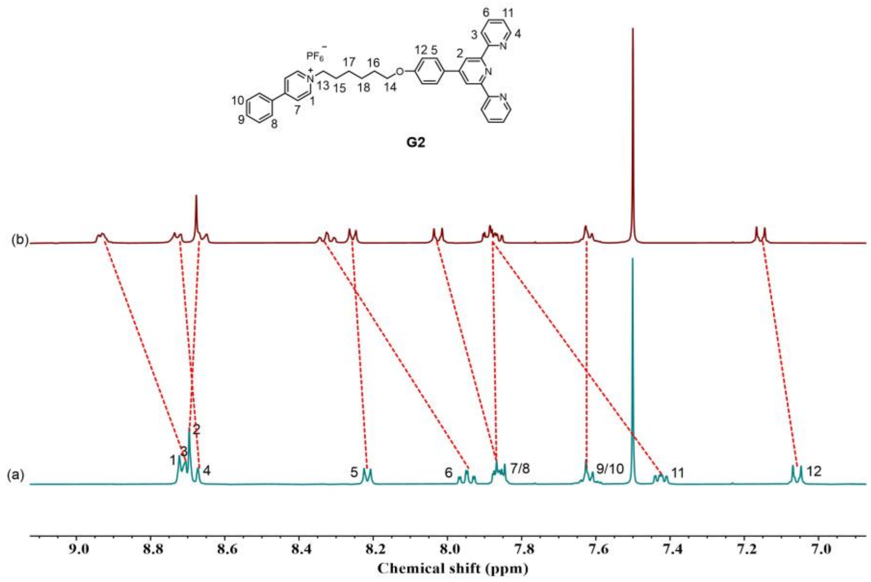

Figure S8 Stacked ^{1}H NMR spectra of (a) **G2**; (b) **G2**:Zn^{2+} = 1:1. (400 MHz, $CDCl_3/CD_3CN$ = 1:1, v/v, 298 K). [Zn^{2+}] = [**G2**] = 1.0 mM.

Job plot for the determination of stoichiometry of host–guest complexes

Job plot obtained by combining host **H1** with **G** (**G1** or **G2**) in mole ratios from 1:0 to 0:1 (total concentration is fixed at 50 μM, solvent is $CHCl_3/CH_3CN$ = 1:1, v/v). The *y*-axis is the fractional decrease in UV absorbance at each composition compared to what would occur if all guests were unbound, that is, it takes account of the varying amount of **G** (**G1** or **G2**). The maximum close to 0.5 is a clear indication of formation of the host–guest complex in 1:1 or n:n stoichiometry.

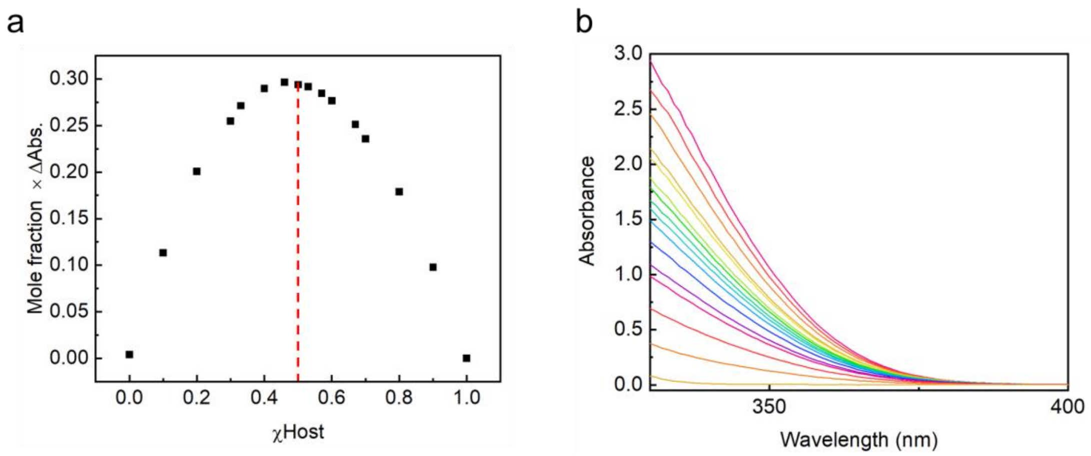

Figure S9 (a) The absorbance at 340 nm is plotted as a function of the **H1** molar ratio. The maximum close to 0.5 is a clear indication of formation of the **H1** ⊃ **G1** complex in 1:1 or n:n

stoichiometry. (b) UV–vis absorption curve profiles of **H1** on addition of increasing amounts of **G1** in $CDCl_3/CD_3CN$ = 1:1, v/v, 298 K.

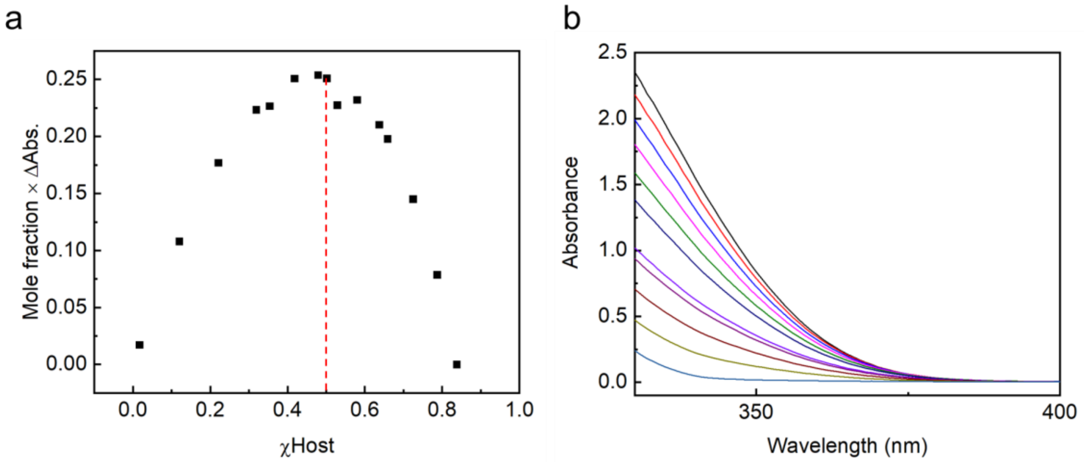

Figure S10 (a) The absorbance at 340 nm is plotted as a function of the **H1** molar ratio. The maximum close to 0.5 is a clear indication of formation of the **H1** ⊃ **G2** complex in 1:1 or n:n stoichiometry. (b) UV–vis absorption curve profiles of **H1** on addition of increasing amounts of **G2** in $CDCl_3/CD_3CN$ = 1:1, v/v, 298 K.

UV–vis titration experiments of macrocycle H1 and guest G2

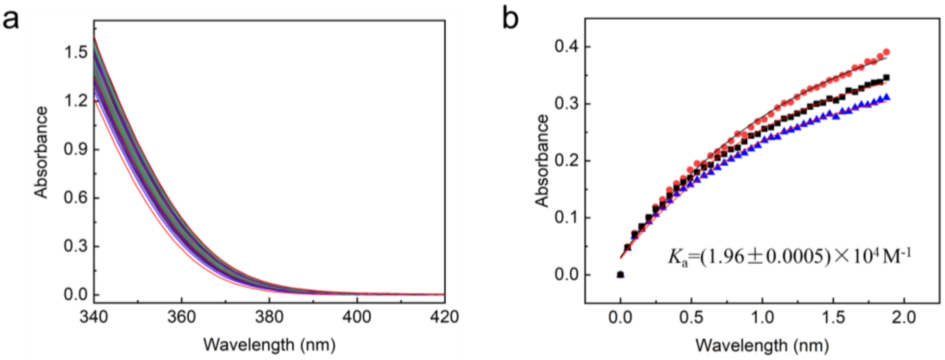

Figure S11 (a) Stacked UV–vis spectra of **H1** (50 μM) titrated with **G2** from 0 equiv to 2.0 equiv in $CHCl_3/CH_3CN$ (1:1, v/v) at 298 K. (b) Curve fitting of the binding constant of **G2** ⊂ **H1** in $CHCl_3/CH_3CN$ (1:1, v/v, 298 K). The reported binding constant is the average value based on fitting of the absorbance at 333 nm, 357 nm, and 365 nm.

HRMS spectra of host–guest complexes H1 + G2

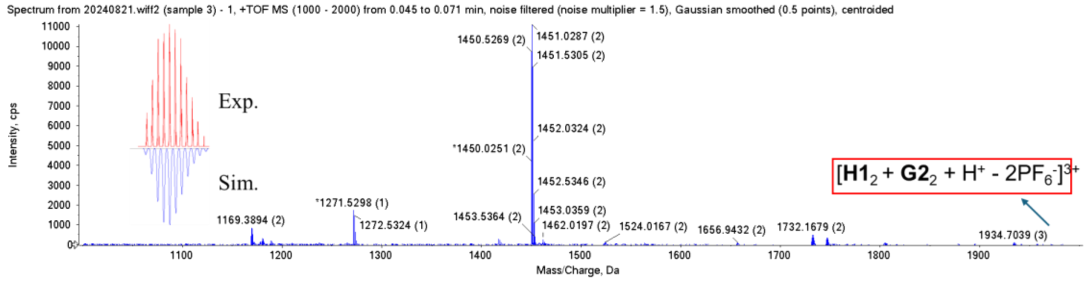

Figure S12 HRMS of **H1** ⊃ **G2** complex in $CHCl_3/CH_3CN$ 1:1, v/v). The calculated (blue) and experimental (red) isotopic distribution for [$\mathbf{H1_2}$ + $\mathbf{G2}_2$ + H^+ − $2PF_6^-$]$^{2+}$, m/z 1933.7006, found 1934.7039.

2D NOESY NMR of the H1 ⊃ G2 complex

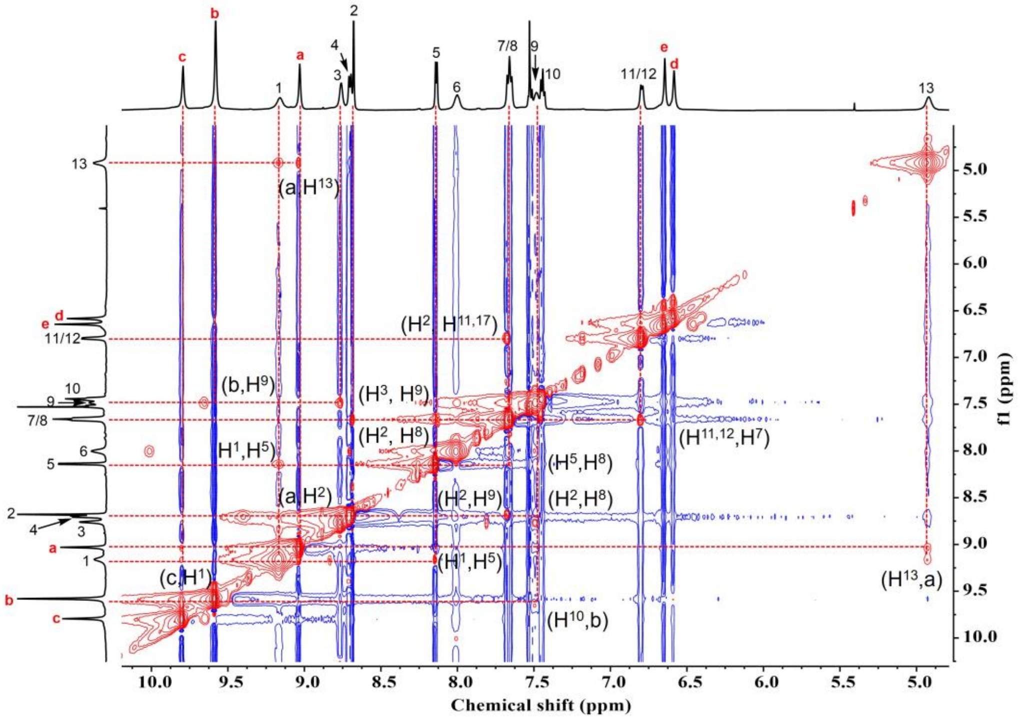

Figure S13 Expanded 2D-NOESY spectrum of **H1** ⊃ **G2**. (600 MHz, $CDCl_3/CD_3CN$= 1:1, v/v, 298 K, mixing time=0.4 s, [**H1**]= [**G2**] = 10 mM)

X-ray crystal structure of G1 ⊂ H2

Crystallographic data (excluding structure factors) for **G1** ⊂ **H2** reported in this communication have been deposited with the Cambridge Crystallographic Data Centre as supplementary publication no. CCDC–2384953. Data collection and structure refinement details can be found in the CIF files or obtained free of charge via www.ccdc.cam.ac.uk/data_request/cif.

Table S1 Crystallographic data and structure refinement for **G1** ⊂ **H2**

Identification code	**G1** ⊂ **H2**
Empirical formula	$C_{96}H_{121}F_6N_8O_{18}P$
Formula weight	1819.98
Temperature/K	123(2)
Crystal system	monoclinic
Space group	P21/c
a/Å	24.434(4)
b/Å	20.026(3)
c/Å	23.779(4)
α/°	90.00
β/°	118.706(2)
γ/°	90.00
Volume/Å3	10205(3)
Z	4
ρcalcg/cm3	1.185
μ/mm-1	0.103
F(000)	3864.0
Crystal size/mm3	0.28 × 0.21 × 0.18
Radiation	Mo Kα (λ = 0.71073)
2Θ range for data collection/°	2.82 to 50.2
Index ranges	-29 ≤ h ≤ 29, -20 ≤ k ≤ 23, -28 ≤ l ≤ 21
Reflections collected	48435
Independent reflections	17763 [Rint = 0.0643, Rsigma = 0.0840]
Data/restraints/parameters	17763/2166/1263
Goodness-of-fit on F2	1.173
Final R indexes [I>=2σ (I)]	R1 = 0.1072, wR2 = 0.2846
Final R indexes [all data]	R1 = 0.1709, wR2 = 0.3153
Largest diff. peak/hole / e Å-3	0.92/-0.52

DLS data of G2 + $Zn(ClO_4)_2$ and H1 + G2 + $Zn(ClO_4)_2$

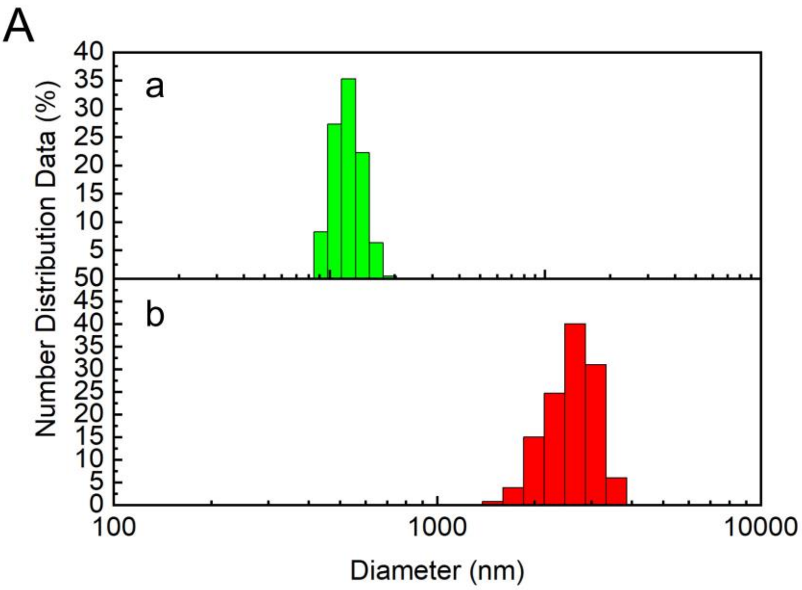

Figure S14 DLS of (a) **G2** + $Zn(ClO_4)_2$; (b) **H1** + **G2** + $Zn(ClO_4)_2$. Solvent: $CHCl_3/CH_3CN$, 1:1, v/v, 298 K.

TEM of H1 + G2 + $Zn(ClO_4)_2$ at variable concentration

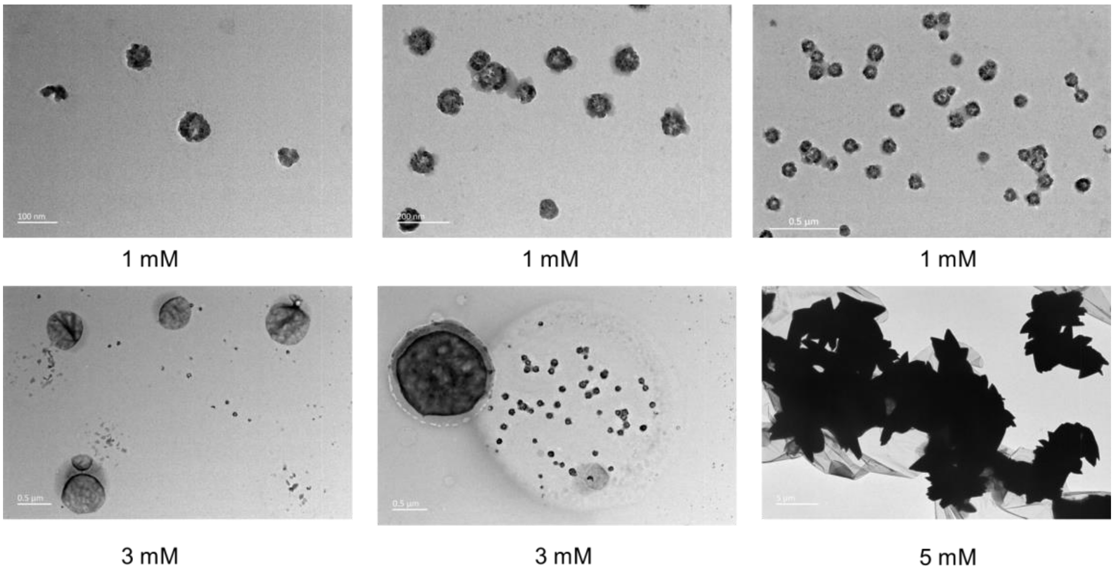

Figure S15 TEM images of **H1**:**G2**:Zn^{2+} = 1:1:1 at different concentrations ($CHCl_3/CH_3CN$ = 1:1, v/v, 298K).

Table S2 Summary of state-of-the-art macrocycle-based binding motifs and their critical polymerization concentrations

Entry	Critical aggregation concentration (CPC)	Solvent	Reference
1	75 mM	CH_3CN	*Angew. Chem. Int. Ed.* **2010**, *49*, 1090 –1094
2	19 mM	$CHCl_3/CH_3CN$ (3:2, v/v)	*Polym. Chem.*, **2013**, *4*, 4292–4297
3	100 mM	CH_3CN	*Polym. Chem.*, **2013**, *4*, 3312–3322
4	9 mM	$CHCl_3/CH_3OH$ (1:1, v/v)	*Chem. Commun.*, **2014**, *50*, 722--724
5	50 mM	CH_3CN	*Dalton Trans.* **2015**, *44*, 20334–20337
6	0.15 mM	$CHCl_3/CH_3CN$ (4:1, v/v)	*Chem. Eur. J.* **2016**, *22*, 6881–6890
7	9 mM	CH_3CN	*J. Photoch. Photobio. A.* **2016**, *331*, 240-246
8	67 mM	$CHCl_3$	*Polym. Chem.*, **2016**, *7*, 5221
9	9 mM	CH_3CN	*Polym. Chem.* **2019**, *10*, 3342
10	5 mM	$CHCl_3$	*J. Am. Chem. Soc.* **2019**, *141*, 4980−4989
11	28 mM	$CHCl_3$	*Chem. Commun.*, **2021**, *57*, 4186–4189
12	22 mM	$CHCl_3/CH_3OH$ (3:1, v/v)	*Polym. Chem.*, **2022**, *13*, 5775–5780
13	17 μM	$CHCl_3/CH_3CN$ (1:1, v/v)	**This work**

References

[1] Wu, J.; Luo, Y.; Chen, L.; Sun, X.; Chen, X.; Qin, S.; Feng, W.; Li, X.; Yuan, L. *Chem. Commun.*, **2022**, *58*, 12867-12870.

[2] Xu, K.; Li, W. J.; Sun, R.; Luo, L. H.; Chen, X.; Zhang, C. C.; Zheng, X. L.; Yuan, M. L.; Fu, H. Y.; Li, R. X. and Chen, H. *Org. Lett.* **2020**, *22*, 6107–6111.

[3] Beloglazkina, E. K.; Manzheliy, E. A.; Moiseeva, A. A.; Maloshitskaya, O. A.; Zyk, N. V.; Skvortsov, D. A.; Osterman, I. A.; Sergiev, P. V.; Dontsova, O. A.; Ivanenkov, Y. A.; Veselov, M. S.; Majouga, A. G., *Polyhedron.,* **2016**, *107*, 27-37.

[4] Loeb, S. J.; Tiburcio, J.; Vella, S. J.; Wisner, J. A. *Org. Biomol. Chem.,* **2006,** *4*, 667-680.

[5] *Gaussian 09,* Revision E.01; Gaussian, Inc.: Wallingford, CT, 2013.
